# DRG2 is required for surface localization of PD-L1 and the efficacy of anti-PD-1 therapy

**DOI:** 10.1038/s41420-024-02027-x

**Published:** 2024-05-27

**Authors:** Seong Hee Choi, Muralidharan Mani, Jeonghwan Kim, Wha Ja Cho, Thomas F. J. Martin, Jee Hyun Kim, Hun Su Chu, Won Jin Jeong, Young-Wook Won, Byung Ju Lee, Byungyong Ahn, Junil Kim, Do Yong Jeon, Jeong Woo Park

**Affiliations:** 1 Department of Biological Sciences, University of Ulsan, Ulsan, Korea; 2 RopheLBio, B102, Seoul Forest M Tower, Seoul, Korea; 3 Department of Biochemistry, University of Wisconsin-Madison, Madison, WI USA; 4 School of System Biomedical Science, Soongsil University, Seoul, Korea; 5 Department of Biomedical Engineering, University of North Texas, Denton, TX USA; 6 Basic-Clinic Convergence Research Institute, University of Ulsan, Ulsan, Korea; 7 Department of Food Science and Nutrition, University of Ulsan, Ulsan, Korea

**Keywords:** Cancer immunotherapy, Endosomes, Tumour biomarkers

## Abstract

More than half of tumor patients with high PD-L1 expression do not respond to anti-PD-1/PD-L1 therapy, and the underlying mechanisms are yet to be clarified. Here we show that developmentally regulated GTP-binding protein 2 (DRG2) is required for response of PD-L1-expressing tumors to anti-PD-1 therapy. DRG2 depletion enhanced IFN-γ signaling and increased the PD-L1 level in melanoma cells. However, it inhibited recycling of endosomal PD-L1 and reduced surface PD-L1 levels, which led to defects in interaction with PD-1. Anti-PD-1 did not expand effector-like T cells within DRG2-depleted tumors and failed to improve the survival of DRG2-depleted tumor-bearing mice. Cohort analysis revealed that patients bearing melanoma with low DRG2 protein levels were resistant to anti-PD-1 therapy. These findings identify DRG2 as a key regulator of recycling of endosomal PD-L1 and response to anti-PD-1 therapy and provide insights into how to increase the correlation between PD-L1 expression and response to anti-PD-1 therapy.

## Introduction

Programmed cell death ligand-1 (PD-L1) on the surface of antigen presenting cells and tumor cells interacts with programmed cell death protein-1 (PD-1) receptor on T cells to inhibit T-cell activity by eliciting immune checkpoint responses [1, 2] Tumor cells escape immune surveillance by upregulating the expression of PD-L1 in response to IFN-γ secreted by activated T cells [3, 4]. Notably, monoclonal antibodies blocking PD-1/PD-L1 interactions demonstrate remarkably durable and persistent responses, with some patients remaining free from cancer progression for many years [5, 6]. Although PD-1/PD-L1 blockade therapies provide a specific and relative safe anti-cancer strategy, many cancer patients fail to respond to PD-1/PD-L1 blockade therapies [7, 8]. The expression of PD-L1 on tumor cells is associated with enhanced response to PD-1/PD-L1 blockade therapies in some tumor types [9]. For instance, patients overexpressing PD-L1 are more likely to show a better prognosis and benefit from PD-1/PD-L1 immune checkpoint blockers (ICBs) [10–12]. Thus, PD-L1 diagnostic immunohistochemistry (IHC) assays have been approved by the US FDA for assessment of PD-L1 expression on tumor cells and are currently used as biomarkers to guide the selection of patients to receive PD-1/PD-L1 ICBs [13, 14]. Despite the association between PD-L1 expression and clinical benefit from ICBs in diverse cancer types, a poor predictive value of PD-L1 expression by IHC has been reported in some cancers such as small cell lung cancer [15, 16], malignant melanoma [17, 18], hepatocellular carcinoma, and renal cell carcinoma [9]. However, in melanoma, multiple studies revealed that PD-L1 expression in cancer cells correlated well with response to ICBs [11, 19, 20] and the FDA approved the 28-8 pharmDx assay as a complementary diagnostic for IHC detection of PD-L1 [21]. This suggest that there might be potential for PD-L1 expression to serve as a predictive biomarker in melanoma patients treated with ICBs. Until now, the underlying mechanisms have been poorly understood, and developing a means to improve the utility of PD-L1 expression as a predictive marker is critical.

Most surface receptors are internalized and sorted into either recycling endosomes or late endosomes [22, 23]. The intracellular trafficking of endosomes is regulated by the Rab family of monomeric GTPases [24]. GTP-bound activated Rab5 functions in the internalization of surface molecules, and the biogenesis of early endosomes [25, 26]. Cargoes in the early endosomes can be sorted to Rab7 endosomes and lysosomes or recycled to the plasma membrane via Rab11-positive recycling endosomes [24, 27]. As receptors move from early endosomes to late endosomes or recycling endosomes, Rab5 should be deactivated and removed from the early endosomes since defects in Rab5 deactivation inhibit the endosomal cargos trafficking from Rab5 early endosomes to Rab7 late endosomes [28, 29] or to Rab11 recycling endosomes [30, 31]. PD-L1 is commonly expressed on the surface of tumor cells [32]. However, a large portion of surface PD-L1 is continuously internalized and recycled back to the surface or degraded by the lysosome pathway [33, 34]. While PD-L1 on the cell surface interacts with PD-1 on the CD8 T cells and suppresses their activity [32, 35], intracellular PD-L1 cannot interact with PD-1 on the CD8 T cells [34].

Developmentally regulated GTP-binding proteins (DRGs) are a novel class of evolutionarily conserved GTP-binding proteins that constitute a subfamily of the GTPase superfamily [36]. Previously, we found that DRG2 interacts with Rab5 on early endosomes, and cells lacking DRG2 show defects in endosomal Rab5 deactivation [37]. We also found that DRG2 depletion causes a delay in recycling of transferrin receptor to the plasma membrane and degradation of epidermal growth factor receptor (EGFR) [37], thus prolonging EGFR localization in Rab5-containing endosomes [38]. Together, these studies suggest that DRG2 may act as a regulator of endosomal trafficking of PD-L1 through regulation of endosomal Rab5 activity. Here, we demonstrate that DRG2 depletion increased expression of PD-L1 in cancer cells but it altered intracellular trafficking of PD-L1 and led to endosomal localization of PD-L1 in cancer cells. The endosomal accumulation of PD-L1 in DRG2-depleted cancer cells limited the response of mice bearing the DRG2-depleted tumor to anti-PD-1 therapy. These results identify DRG2 as a key determinant for response of PD-L1-expressing cancer cells to anti-PD-1 therapy through regulation of intracellular localization of PD-L1 in cancer cells and as a novel therapeutic target to overcome PD-1/PD-L1-mediated immune escape of cancer cells.

## Results

### DRG2 depletion in melanoma cells enhances both the PD-L1 expression in tumor cells and the proportion of IFN-γ-expressing CD8 T cells in tumor-infiltrating immune cells

**Fig. 1:**
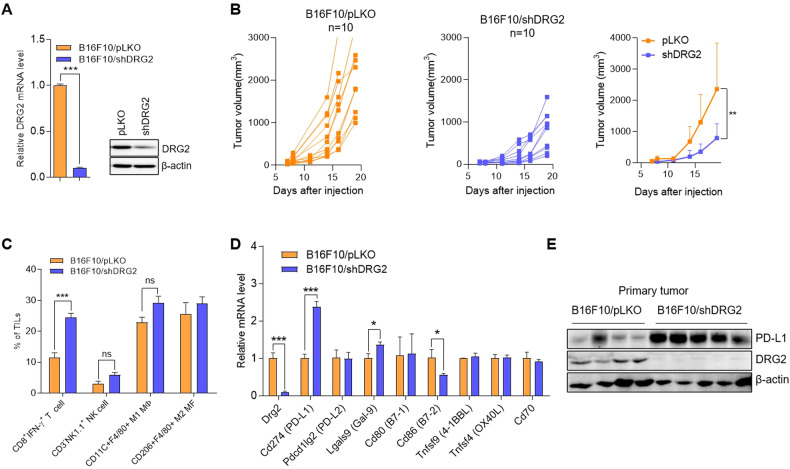
DRG2 depletion in melanoma cells enhances PD-L1 expression in cancer cells but increases the proportion of tumor-infiltrating CD8 T cells and inhibits tumor growth. **A** Confirmation of DRG2 depletion in B16F10/shDRG2 cells by qRT-PCR and western blot analysis. **B** Individual and mean tumor growth curves for mice s.c. injected with B16F10/pLKO or B16F10/shDRG2 cells. Data are pooled from two independent experiments (*n* = 10 per group). The data are expressed as means ± SD. Two-way ANOVA, **P < 0.01. **C**–**E** Tumor masses were collected 15 days after s.c. injection of melanoma cells. **C** FACS analysis for immune cells in TIICs of B16F10/pLKO and B16F10/shDRG2 tumors. Graph represents % of immune cells within TIICs. Values are the mean ± SD of two independent experiments (*n* = 3 per group per experiment). Student’s *t* test. ***P < 0.001. ns not significant. See also Supplementary Fig. S1. **D** qRT-PCR analysis for expression of immune checkpoint molecules in B16F10/pLKO and B16F10/shDRG2 tumors. Values are the mean ± SD of two independent experiments (*n* = 3 per group per experiment). Student’s *t* test. *P < 0.05; ***P < 0.001. **E** Western blot analysis for PD-L1 in B16F10/pLKO and B16F10/shDRG2 tumors.

Previously, we reported that expression of DRG2 is significantly higher in human metastatic melanoma than primary melanoma and dysplastic nevi and that DRG2 depletion in melanoma cells inhibits melanoma growth and metastasis in mice [39]. Tumor-infiltrating immune cells(TIICs) influence the anti-tumor immune response and tumor growth [40, 41]. Here, we analyzed whether DRG2 depletion in melanoma cells affects components of the TIICs, leading to inhibition of tumor growth. Consistent with our previous report [39], mice subcutaneously (s.c.) injected with DRG2-depleted B16F10 (B16F10/shDRG2) cells (Fig. 1A) developed small tumors compared with those s.c. injected with control B16F10 (B16F10/pLKO) cells (Fig. 1B). Phenotypic analysis of TIICs revealed that (while there was no significant difference in the number of NK cells, M1, and M2 macrophages between DRG2-depleted tumors and wild-type tumors) the number of IFN-γ-expressing CD8^+^ T cells was significantly increased in DRG2-depleted tumor compared with wild-type tumors (Fig. 1C and Supplementary Fig. S1). These data suggest that DRG2 depletion in cancer cells increases the proportion of activated CD8^+^ T cells in the TIICs, which plays a crucial role in anti-tumor immunity. The anti-tumor response of CD8^+^ T cells in the tumor microenvironment is regulated by multiple immune-checkpoint receptors and ligands upregulated in various types of tumors [9, 42]. We next investigated the effect of DRG2 depletion on the expression of immune-checkpoint molecules in melanoma tumors. While DRG2 depletion did not affect the expression of *Pdcd1lg2* (*PD-L2*), *Cd80* (*B7-1*), *Tnfsf9* (*4-1BBL*), *Tnfsf4* (*OX40L*), and *Cd70*, it decreased *Cd86* (*B7-2*) expression but increased the expression of *Lgals9* (*Gal-9*) and *Cd274* (*PD-L1*) (Fig. 1D). Since the most dramatically changed gene expression was observed in *PD-L1*, we focused on the effect of DRG2 depletion on the expression of *PD-L1*. The expression level of PD-L1 significantly increased in DRG2-depleted tumors compared with that of wild-type tumors (Fig. 1E and Supplemental Material 1).

### RNA-Seq transcriptome analysis reveals enhanced IFN-γ signaling pathways in DRG2-depleted B16F10 cells

**Fig. 2:**
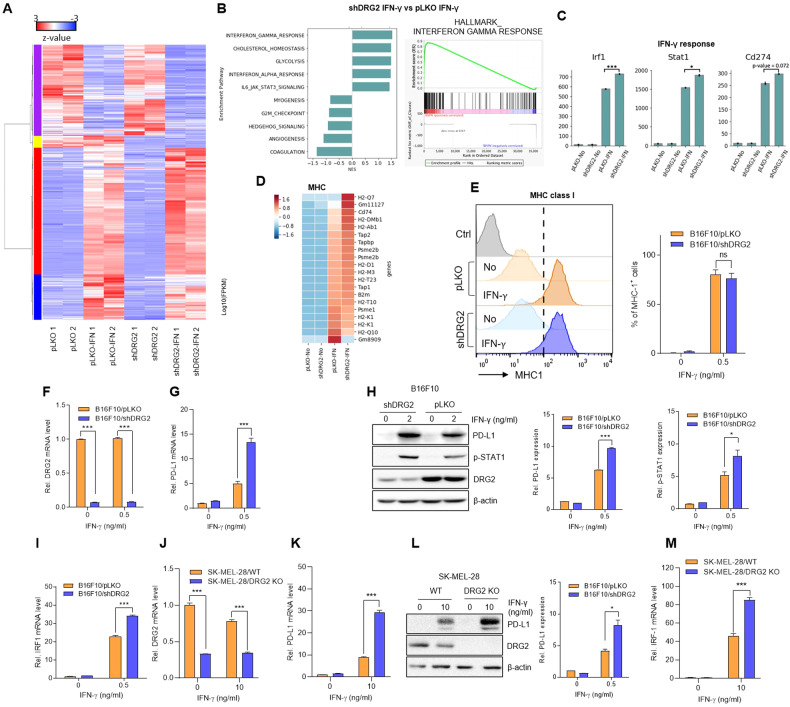
DRG2 deficiency enhances IFN-γ responses and PD-L1 expression in melanoma cells. **A**–**D** RNA-Seq analysis. **A** Heatmap of 500 DEGs in non-treated and IFNγ-treated B16F10/pLKO and B16F10/shDRG2 cells. **B** Gene set enrichment analysis (GSEA). Bar plot of enriched GSEA pathways in IFNγ-treated B16F10/pLKO cells vs. IFNγ-treated B16F10/pLKO cells. Enrichment plot of IFNγ responses positively enriched in IFNγ-treated B16F10/shDRG2 cells. **C** Expression of genes involved in IFNγ response in non-treated and IFNγ-treated B16F10/pLKO and B16F10/shDRG2 cells. The y-axis corresponds to fragments per kilobase of exon model per million mapped reads (FPKM) measured by RNA-Seq (EdgeR at FDR < 0.05). Student’s *t* test. *P < 0.05; ***P < 0.001. See also Supplementary Fig. S2. **D** Heatmap of MHC genes in non-treated and IFNγ-treated B16F10/pLKO and B16F10/shDRG2 cells. **E**–**I** B16F10/pLKO and B16F10/shDRG2 cells were treated with indicated concentration of IFNγ for 24 h. **E** FACS analysis for MHC class I expression in IFNγ-treated B16F10/pLKO and B16F10/shDRG2 cells. Flow cytometry graphs and quantification of the proportion of cells showing IFNγ-induced expression of MHC class I (dotted line). **G** qRT-PCR analysis for PD-L1. **H** Representative Western blot images and relative densitometric bar graphs of PD-L1 and phosphorylated STAT1. Fold change in expression levels was calculated relative to the values of non-treated B16F10/pLKO cells. **I** qRT-PCR analysis for *IRF1*. **J**–**M** SK-MEL-28/WT and SK-MEL-28/DRG2 KO human melanoma cells were treated with 10 ng/ml IFNγ for 24 h. **J** qRT-PCR analysis for DRG2. **K** qRT-PCR analysis for *PD-L1*. **L** Representative Western blot images and relative densitometric bar graphs of PD-L1. Fold change in expression levels was calculated relative to the values of non-treated wild-type SK-MEL-28 cells. **M** qRT-PCR analysis for *IRF1*. Values are the mean ± SD of two independent experiments (*n* = 3 per group per experiment). Student’s *t* test. *P < 0.05; ***P < 0.001. ns not significant. See also Supplementary Fig. S3.

In the tumor microenvironment, cancer cells upregulate the expression of *PD-L1* in response to IFN-γ secreted by activated T cells [3, 4, 32]. To examine the effect of DRG2 depletion on global transcription response to IFN-γ, we conducted RNA-seq analysis using RNA isolated from B16F10/pLKO and B16F10/shDRG2 cells at 24 h after IFN-γ treatment. In our comparison of IFN-γ-treated B16F10/pLKO cells with non-treated B16F10/pLKO cells, 1192 DEGs were identified, with 852 upregulated and 340 downregulated DEGs (adjusted *P* value < 0.05 with an absolute log2FC > 1.5) (Supplementary Table S1), as shown in the volcano plot (Supplementary Fig. S2A). Similarly, in our comparison of IFN-γ-treated B16F10/shDRG2 cells with non-treated B16F10/shDRG2 cells, 1378 DEGs were identified, with 909 upregulated and 469 downregulated DEGs (Supplementary Fig. S2B and Supplementary Table S2). In our comparison of IFN-γ-treated B16F10/shDRG2 cells with IFN-γ-treated B16F10/pLKO cells, 153 DEGs were identified, with 33 upregulated and 120 downregulated DEGs (adjusted *P* value < 0.05 with an absolute log2FC > 1) (Supplementary Table S3). Two-way unsupervised hierarchical clustering of the union of the DEGs showed a clear separation of IFN-γ-treated B16F10 cells from non-treated B16F10 cells and a coherent pattern between IFN-γ-treated B16F10/pLKO cells and B16F10/shDRG2 cells (Fig. 2A and Supplementary Fig. S2C). The volcano plots and gene set enrichment analysis (GSEA) showed that the IFN-γ response was significantly upregulated in both IFN-γ-treated B16F10/pLKO cells (Supplementary Fig. S2A, D) and B16F10/shDRG2 cells (Supplementary Fig. S2B, E).

We focused on the DEGs between IFN-γ-treated B16F10/pLKO and B16F10/shDRG2 cells to study the potential function of DRG2 in IFN-γ response. GSEA showed that the IFN-γ response was significantly positively enriched in B16F10/shDRG2 cells (Fig. 2B). IFN-γ upregulated several genes involved in the IFN-γ response, including *Irf1*, *Stat1*, and *Cd274* (*PD-L1*) in B16F10 cells and their induced levels were significantly higher in B16F10/shDRG2 cells than B16F10/pLKO cells (Fig. 2C and Supplementary Fig. S2F). It is well known that MHC antigen processing and presentation are upregulated by IFN-γ [43]. IFN-γ consistently upregulated the expression of MHC molecules in both B16F10/pLKO and B16F10/shDRG2 cells (Fig. 2D). These results indicate that DRG2 depletion does not inhibit (but rather significantly enhances) the IFN-γ response in cancer cells. In the tumor microenvironment (TME), IFNγ stimulates cancer cells to induce EMT [44], which can contribute to immunosuppression [45, 46] and to resistance to ICBs [47]. However, IFN-γ did not induce EMT-related genes in both B16F10/pLKO and B16F10/shDRG2 cells (Supplementary Fig. S2G).

We next confirmed RNA-Seq results using FACS, qPCR and western blots. Consistent with RNA-Seq results, FACS analysis revealed enhanced expression of MHC class I in both B16F10/pLKO and B16F10/shDRG2 cells after IFN-γ treatment (Fig. 2E). In addition, DRG2 depletion using either shRNA (Fig. 2F) or siRNA (Supplementary Fig. S3A) significantly enhanced the expression of PD-L1 in B16F10 cells compared with wild-type B16F10 cells (Fig. 2G, H, Supplementary Fig. S3B, C and Supplemental Materials 2A and 3). IFN-γ induces the expression of PD-L1 in cancer cells through JAK1/2–STAT1/2/3–IRF1 pathway [32, 48]. We found that *DRG2* depletion using either shRNA or siRNA increased the phosphorylation of STAT1 (Fig. 2H and Supplementary Fig. S3C, and Supplemental Material 2A) and (in turn) the expression level of *IRF1* in B16F10 (Fig. 2I and Supplementary Fig. S3D) after IFN-γ treatment. We also found that DRG2 depletion significantly increased the expression of PD-L1 and *IRF1* in SK-MEL-28 human melanoma cells (Fig. 2J–M and Supplemental Material 2B) and CT26 murine colorectal carcinoma cells (Supplementary Fig. S3E–G). These results indicate that, even though we did not provide detail mechanisms, DRG2 depletion enhances IFN-γ signaling pathway and in turn increases PD-L1 expression in cancer cells.

### PD-L1 in the DRG2-depleted cancer cells shows defects in binding with PD-1 and in suppressing T cell activity

**Fig. 3:**
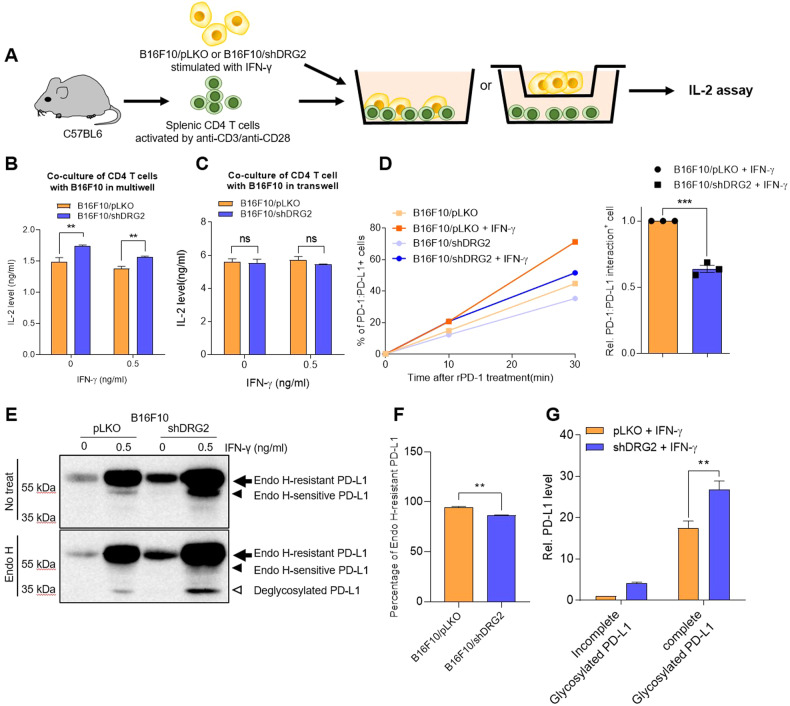
DRG2-depleted melanoma cells with high PD-L1 level show defects in interactions with recombinant PD-1 and inhibition of T cells activity. **A**–**C** DRG2-depleted melanoma cells show defects in inhibiting T cells activity. **A** Scheme of co-culture experiment using splenic CD4 T cells and B16F10/pLKO or B16F10/shDRG2. IL-2 levels determined by ELISA in the supernatant of cells co-cultured in (**B**) 96-well plate or **C** transwell plates. Values are the mean ± SD of two independent experiments (*n* = 3 per group per experiment). Student’s *t* test. **P < 0.01. ns not significant. **D** FACS analysis for cell-bound recombinant PD-1. Values represent % of PD-1-positive cells and are presented as mean ± SD of two independent experiments (*n* = 3 per group per experiment). Student’s *t* test. **P < 0.01. ns not significant. See also Supplementary Fig. S4. **E** Representative image of western blot analysis of Endo H-treated cell lysates from IFN-γ-treated B16F10/pLKO and B16F10/shDRG2 cells. Arrow, endo H-resistant PD-L1; filled arrowhead, endo-H-sensitive PD-L1; open arrowhead, deglycosylated PD-L1. **F** Graph represents percentage of endo H-resistant PD-L1. **G** Graph represents PD-L1 levels relative to the levels obtained from Endo H-sensitive PD-L1 of B16F10/pLKO (means ± SD of three independent experiments). **P < 0.01.

It is well-known that PD-L1 on cancer cells interact with PD-1 on T cells and inhibits the activity of tumor-infiltrating T cells [1, 2]. It is interesting to observe the increase in IFN-γ-expressing CD8^+^ T cells within DRG2-depleted tumors with increased PD-L1 expression. We thus determined whether DRG2-depleted cancer cells with enhanced PD-L1 expression have defects in suppressing T cell activity by co-culturing mouse splenic CD4^+^ T cells with IFN-γ-stimulated B16F10 cells (Fig. 3A). CD4^+^ T cells co-cultured with B16F10/shDRG2 melanoma cells produced significantly higher levels of IL-2 than those co-cultured with B16F10/pLKO (Fig. 3B). When the CD4^+^ T cells were co-cultured with B16F10 cells using a transwell culture system that prohibits the direct cell-to-cell contact (Fig. 3A), we could not detect any difference in the IL-2 secretion between the CD4^+^ T cells co-cultured with B16F10/pLKO and B16F10/shDRG2 cells (Fig. 3C). These results suggest that PD-L1 in DRG2-depleted B16F10 cells may have defects when inhibiting T cell activity. We next used recombinant PD-1 to determine whether PD-L1 molecules of the DRG2-depleted cells bind equally well with PD-1 as those of the wild-type cells. B16F10/shDRG2 cells exhibited less PD-1 protein binding to the cell surface than B16F10/pLKO cells as determined by FACS analysis (Fig. 3D and Supplementary Fig. S4). Collectively, all these results suggested that PD-L1 in DRG2-depleted B16F10 cells have functional defects in binding with PD-1 and, thus, in suppressing T cell activity.

### DRG2 depletion does not reduce the level of PD-L1 with N-linked glycan maturation in cancer cells

PD-L1 is a glycoprotein with four N-linked glycan [49], and glycosylation of PD-L1 is required for interaction with PD-1 and its immunosuppressive function [50]. We previously reported that DRG2 depletion induces Golgi fragmentation [38]. Thus, it is possible that DRG2 depletion may result in defective N-linked glycosylation of PD-L1 and defects in binding with PD-1. The defect in N-linked glycan maturation of PD-L1 may increase the sensitivity to endoglycosidase H (Endo H). To test this, we treated lysates of IFN-γ-stimulated melanoma cells with Endo H and compared Endo H-sensitivity of their PD-L1. Even though B16F10/shDRG2 cells contained a lower percentage of Endo H-resistant PD-L1 than B16F10/pLKO cells (Fig. 3E, F and Supplemental Material 4), the former contained significantly higher levels of Endo H-resistant PD-L1s than the latter (Fig. 3E, G and Supplemental Material 4). These results indicate that DRG2 depletion does not reduce the level of PD-L1 with N-linked glycan maturation in cancer cells.

### DRG2 depletion inhibits the endosomal trafficking of PD-L1 and increases the internalized form of PD-L1 in cancer cells

**Fig. 4:**
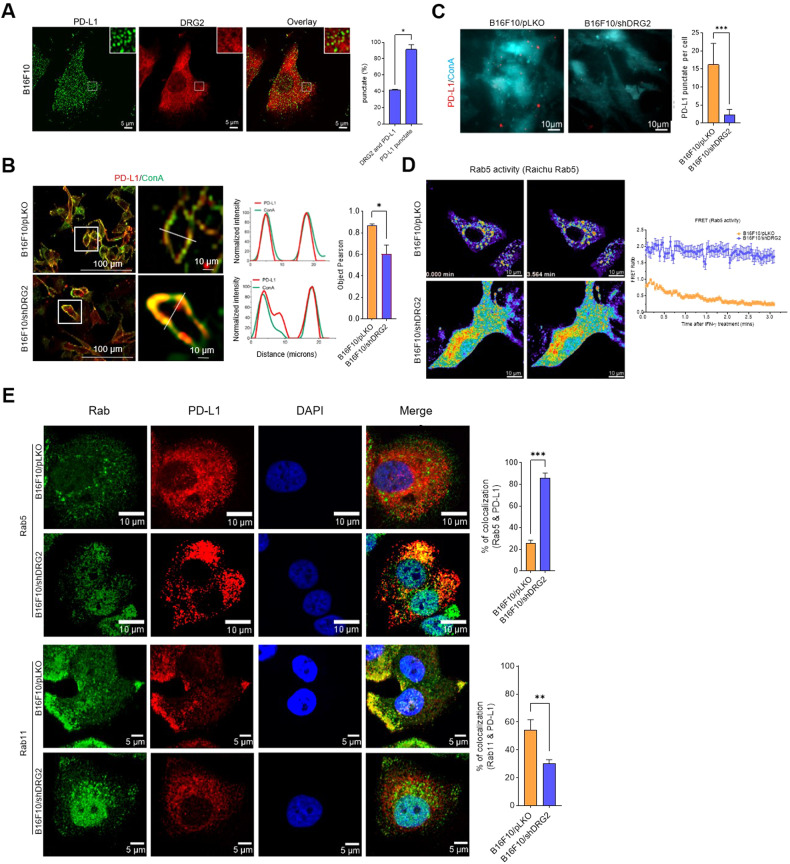
DRG2-depleted melanoma cells show defects in recycling of endosomal PD-L1 and accumulation of PD-L1 at Rab5-endosomes. **A** Representative confocal images of B16F10 cells incubated with anti-DRG2 and anti-PD-L1 at 24 h after IFN-γ treatment. Graph represents % of DRG2-containing puncta among PD-L1-puncta. **B** Representative confocal images of B16F10/pLKO and B16F10/shDRG2 cells incubated with ConA and anti-PD-L1 antibody after IFN-γ treatment. Line graphs represent linear pixel values across cells. Bar graph represents PD-L1 colocalization with ConA in terms of the Pearson’s correlation coefficient (mean ± SD from two experiments, with 20 different cells per group per experiment). *P < 0.05. **C** Representative TIRF images of B16F10/pLKO and B16F10/shDRG2 cells incubated with ConA and anti-PD-L1 antibody after IFN-γ treatment. Bar graph represents number of PD-L1 puncta per cell in the TIRF images (mean ± SD from two experiments, with 20 different cells per group per experiment). ***P < 0.001. **D** FRET images of B16F10/pLKO and B16F10/shDRG2 cells expressing Raichu-Rab5 at indicated time point after IFN-γ treatment. Graph shows FRET/CFP ratio at indicated times. **E** Representative confocal images of B16F10/pLKO and B16F10/shDRG2 cells incubated with anti-DRG2, anti-PD-L1, anti-Rab5, and anti-Rab11 at 30 min after IFN-γ treatment. Blue, DAPI staining. Graph represents Pearson’s *R*(*r*) between PD-L1 and Rab5 or Rab11 (mean ± SD from two experiments, with 20 different cells per group per experiment). **P < 0.01; ***P < 0.001. See also Supplementary Fig. S5.

PD-L1 on the surface membrane is continuously internalized to early endosomes and recycled back to the surface by recycling endosomes [33, 34]. We previously reported that DRG2 is located on Rab5-containing early endosomes and that DRG2 depletion blocks endosomal recycling [37]. We also observed the endosomal localization of both PD-L1 and DRG2, even though PD-L1 mostly did not co-localize with DRG2 on the endosomes (Fig. 4A). Considering the continuous internalization and recycling of PD-L1 within endosomes [33, 34], it is possible that DRG2 depletion in cancer cells may block the recycling of internalized PD-L1 and thus reduce the portion of cell surface PD-L1. To confirm this, the surfaces of B16F10/pLKO and B16F10/shDRG2 cells were labelled with *concanavalin* A (Con A) and incubated with PD-L1-specific antibodies. When evaluated by confocal microscopy, PD-L1 in B16F10/shDRG2 cells was less co-localized with ConA than that in B16F10/pLKO cells (Fig. 4B). The PD-L1 of DRG2-depleted SK-MEL-28 human melanoma cells also showed reduced colocalization with Con A (Supplementary Fig. S5A). We also applied total internal reflection fluorescence microscopy (TIRF) to visualize PD-L1 located at or near the plasma membrane. Consistent with results obtained from confocal microscopy, the TIRF image showed a significantly reduced level of PD-L1 in B16F10/shDRG2 compared with B16F10/pLKO (Fig. 4C). These results suggest that recycling of internalized PD-L1 to the cell surface is inhibited in DRG2-depleted cells.

Deactivation of the Rab5 in the early endosome is required to allow endosomal recycling [51], and defects in Rab5 deactivation interfere with endosomal recycling [30, 31]. Previously, we reported that DRG2-depleted cells showed defects in Rab5 deactivation and endosome recycling [37]. Here, we also confirmed that DRG2-depleted cells showed enhanced Rab5 activity in endosomes before and after IFN-γ treatment (Fig. 4D). We next examined the effect of DRG2 depletion on endosomal trafficking of PD-L1 by analyzing colocalization between PD-L1 and endosomal markers (Rab5 and Rab11 for early and recycling endosomes, respectively) using confocal microscopy. Localization of PD-L1 on Rab5 early endosomes was significantly enhanced in B16F10/shDRG2 cells compared with B16F10/pLKO cells (Fig. 4E, top). In contrast, the PD-L1 in B16F10/shDRG2 cells showed significantly reduced colocalization with Rab11 recycling endosomes compared with that in B16F10/pLKO cells (Fig. 4E, bottom). We also found that DRG2 depletion significantly increased the localization of PD-L1 on Rab5 endosome but decreased it on Rab11 endosomes in SK-MEL-28 human melanoma cells (Supplementary Fig. S5B). Collectively, our results suggest that DRG2 is required for endosomal trafficking of PD-L1 and DRG2 depletion blocks the recycling of internalized PD-L1, leading to a decrease in the cell surface PD-L1.

### DRG2-depleted tumors are resistant to anti-PD-L1 therapy

**Fig. 5:**
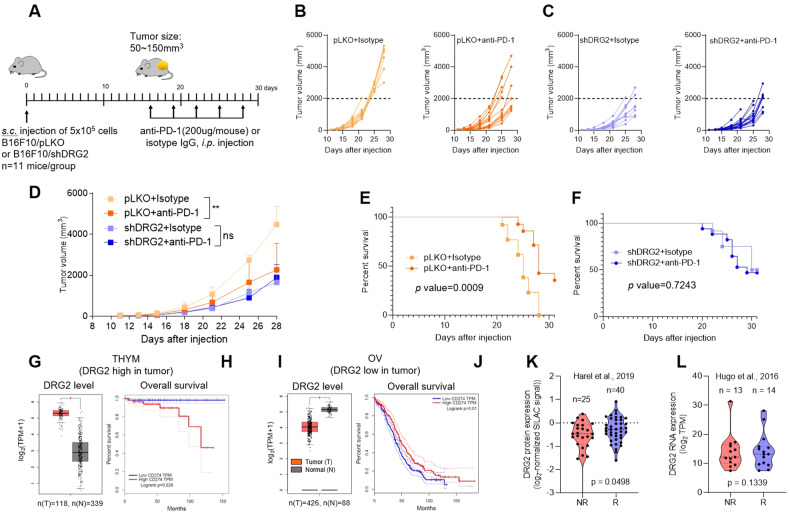
DRG2 deficiency in cancer cells hinders the anti-tumor activity of anti-PD-1 immunotherapy. **A** Schematic representation of the treatments applied to mice that were subcutaneously (s.c.) engrafted with B16F10 melanoma cells. **B**, **C** Individual growth of tumors over time in (**B**) B16F10/pLKO or (**C**) B16F10/shDRG2 tumor-containing mice treated with negative control (isotype) or anti-PD-1. Arrows represents anti-PD-1 treatment. **D** Average tumor sizes from experiment shown in (**B**, **C**). The data are expressed as means ± SD. Two-way ANOVA, **P < 0.01. **E**, **F** Overall survival of (**E**) B16F10/pLKO or (**F**) B16F10/shDRG2 tumor-containing mice treated with IgG or anti-PD-1. *P* values were calculated using a two-sided log-rank test. **G**–**J** Kaplan–Meier survival curves of cancer patients in TCGA according to DRG2 and PD-L1 expression. **G**, **I** Boxplots for DRG2 expression level in terms of log2(TPM + 1) in the tumor (red) and normal (grey) samples and **H**, **J** Kaplan–Meier overall survival curve of (**G**, **H**) thymoma (THYM) patients with high DRG2 expression in tumors and **I**, **J** ovarian serous cystadenocarcinoma (OV) patients with low DRG2 expression in tumors. For Kaplan–Meier plots, patients were split into two equal-sized groups with the median of CD274 (PD-L1) level as the cutoff. One-way ANOVA was used to assess the difference in DRG2 expression. *P < 0.05. Log-rank tests were used to assess the difference in patient survival times between the two groups. **K**, **L** Relationship between (**K**) protein and (**L**) mRNA expression level of DRG2 and anti-PD-1 responses in the anti-PD-1 response cohorts. The middle line in the graph is the mean. To assess the differences in DRG2 expression between anti-PD-1 responding (R) and non-responding groups (NR) robustly, the Student *t* test was used, and the *P* value from a permutated random distribution is shown on the plot.

It has been reported that PD-1/PD-L1 ICBs are efficient for PD-L1 on surface membranes [32, 34]. Thus, we hypothesized that a decrease in the cell surface PD-L1 in DRG2-depleted cancer cells might reduce the therapeutic efficacy of PD-1/PD-L1 ICBs. To test this, we treated syngeneic mice containing established B16F10/pLKO or B16F10/shDRG2 tumors with anti-PD-1 antibody or negative control (isotype IgG), and we monitored the changes in tumor growth and mice survival (Fig. 5A). As expected from our earlier observations, DRG2 depletion impaired the anti-tumor effect of anti-PD-1 antibody (Fig. 5B–F). While treatment of anti-PD-1 antibody markedly retarded tumor progression (Fig. 5B, D) and significantly improved overall survival (Fig. 5E) in mice bearing B16F10/pLKO tumors, it did not affect both tumor progression (Fig. 5C, D) and overall survival (Fig. 5F) in mice bearing B16F10/shDRG2 tumors. These data indicate that DRG2 is required for the therapeutic efficacy of anti-PD-1 antibody.

### DRG2 protein expression shows correlation with response of melanoma patients to anti-PD-1 immunotherapy

To evaluate our findings in a clinical database, we analyzed the expression profile of DRG2 in human tumor samples and paired normal tissues using the Gene Expression Profiling Interactive Analysis (GEPIA) database and its online analysis tool. We selected 2 groups of human tumors: group 1 (thymoma) with higher DRG2 expression in tumors than paired normal tissues (Fig. 5G) and group 2 (ovarian carcinoma) with lower DRG2 expression in tumors than paired normal tissues (Fig. 5I). In each group, patients were split into two equal-sized groups with the median CD274 (PD-L1) level as the cutoff, and we validated the correlation between PD-L1 expression and prognosis. In tumor patients of group 1, the median overall survival (OS) of tumor patients with high PD-L1 expression was significantly shorter than those with low PD-L1 expression (Fig. 5H). However, in tumor patients of group 2, high PD-L1 expression was correlated with enhanced survival outcomes (Fig. 5J).

We next assessed the potential relevance of DRG2 expression in clinical response of melanoma patients to anti-PD-1 therapy using the Hugo cohort [48] and the Harel cohort [52]. While the Harel cohort [52] provides information about DRG2 protein level, the Hugo cohort [53] provided DRG2 mRNA level. High levels of DRG2 protein were associated with increased clinical response to anti-PD-1 therapy in Harel cohort [52] (Fig. 5K). However, DRG2 RNA levels in Hugo cohort [53] did not show such a correlation (Fig. 5L). Even though additional cohorts are necessary to validate these findings, the current data suggest a potential correlation between DRG2 protein levels and tumor response to anti-PD-1 therapy.

### Single-cell profiling of cancer cells after anti-PD-1 treatment

**Fig. 6:**
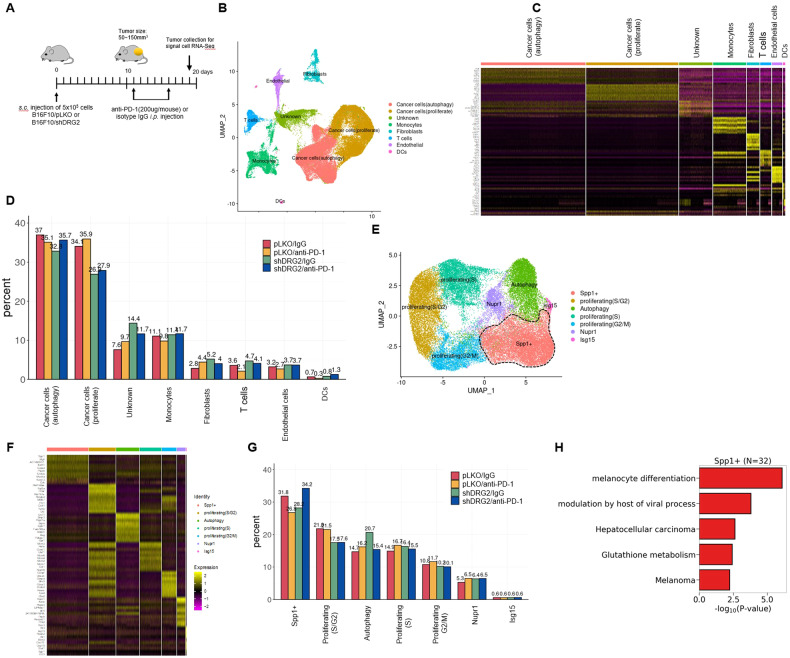
Single-cell RNA-Seq analysis reveals subcluster of DRG2-depleted melanoma cells resistant to anti-PD-1 immunotherapy. **A** Schematic representation of the treatments applied to mice that were subcutaneously (s.c.) engrafted with B16F10 melanoma cells. **B**–**D** Profiling single cells in B16F10/pLKO or B16F10/shDRG2 tumors treated with negative control (IgG) or anti-PD-1. **B** Uniform manifold approximation and projection (UMAP) map of 62,956 cells color-coded for the indicated cell type. **C** Relative expression of the top-10 most differentially expressed genes for each cell clusters. Cluster-defining genes are shown in Supplementary Fig. S6. **D** Relative contribution of each cell cluster (in %) in B16F10/pLKO or B16F10/shDRG2 tumors treated with negative control (IgG) or anti-PD-1. **E** UMAP of 41,412 cancer cells showing 7 subclusters of cancer cells indicated by the color-coded legend. **F** A heatmap based on relative expression of the top-10 most differentially expressed genes for each cell subclusters. Subcluster-defining genes are shown in Supplementary Fig. S7. **G** Relative contribution of each cancer cell subcluster (%) in B16F10/pLKO or B16F10/shDRG2 tumors treated with negative control (IgG) or anti-PD-1. **H** Pathway analysis of the top five molecular signatures distinguishing cancer cell “Spp1 + ” subcluster.

To characterize the response of cancer cells and TIICs in melanoma undergoing anti-PD-1 therapy, we collected tumor masses on day 3 after the second anti-PD-1 treatment, and subjected tumor masses to single-cell RNA sequencing (scRNA-seq) (Fig. 6A). In total, 62,956 cells passed quality control and were carried forward for analyses. We confirmed that there was no cluster bias in IgG- versus anti-PD-1-treated tumor samples or wild-type versus DRG2-depleted B16F10 tumors (Supplementary Fig. S6A). Anchor-based integration of cells from four samples followed by Louvain clustering [54] identified 8 cell types: autophagic cancer cells, proliferating cancer cells, monocytes, fibroblasts, T cells, endothelial cells, dendritic cells (DCs), and unknown cells (Fig. 6B). The 8 cell types were annotated based on the differentially expressed markers: *Bnip3* for autophagic cancer cells, *Mki67* for proliferating cancer cells, *Cd14*, *Apoe*, *C1qc*, *Mrc1*, *Lyz2* for monocytes, *Col3a1* for fibroblasts, *Cd3e* and *Gzma* for T cells, *Flt1* for endothelial cells, and *Klk1* for DCs (Fig. 6C and Supplementary Fig. S6B, C). Consistent with Fig. 1B, C, in tumor mass treated with IgG, cancer cells were less frequent but T cells were more frequent in B16F10/shDRG2 tumors than B16F10/pLKO tumors (Fig. 6D). We evaluated the transcriptional programming of IgG- and anti-PD-1-treated cancer cells (proliferating and autophagic cancer cells; *n* = 41,412). We confirmed the DRG2 depletion in B16F10/shDRG2 cancer cells (Supplementary Fig. S7A). EMT in tumor cells is linked to resistance to ICBs [45–47]. However, we could not find any difference in the expression of genes involved in the EMT among IgG- and αPD-1–treated B16F10/pLKO and B16F10/shDRG2 cells (Supplementary Fig. S7B).

Refined clustering of all B16F10 cancer cells identified 7 subclusters (Fig. 6E–G). All 7 subclusters were found in both wild-type and DRG2-depleted B16F10 tumors treated with either IgG or anti-PD-1 (Fig. 6G). The most dominant subcluster was characterized by expression of “secreted phosphoprotein 1 (Spp1)+” (26.8–34.2% of total cancer cells) (Fig. 6G). Spp1 is highly expressed in human melanoma [55] and plays an important role in proliferation, invasion, and metastasis of cancer cells [56, 57]. In B16F10/pLKO tumors in which progression was inhibited by anti-PD-1 treatment (Fig. 5B, D), anti-PD-1 treatment decreased the percentage of cancer cells in subcluster “Spp1 + ”, while it did not decrease the percentage of cancer cells in other subclusters (Fig. 6G), suggesting that cancer cells in subcluster “Spp1 + ” are anti-PD-1 responders. However, in B16F10/shDRG2 tumors, anti-PD-1 treatment did not decrease the percentage of cancer cells in subcluster “Spp1 + ” but increased it (Fig. 6G). The pseudo-bulk analysis also suggested that the Spp1 expression was significantly downregulated by anti-PD-1 treatment in B16F10/pLKO tumors, whereas it was significantly upregulated by anti-PD-1 treatment in B16F10/shDRG2 tumors (adjusted *P* value is lower than the minimum threshold; Supplementary Fig. S7C). Even though anti-PD-1 treatment decreased the percentage of cancer cells in subcluster “Autophagy” in DRG2-depleted tumors (Fig. 6G), this did not lead to inhibition of the tumor progression (Fig. 5C, D, F), possibly because of expansion of the subcluster “Spp1 + ” population. We next searched KEGG pathways and GO terms associated with highly expressed genes in subcluster “Spp1 + ”, which revealed that subcluster “Spp1 + ” was distinguished by upregulation of genes involved in the melanocyte differentiation and melanoma (Fig. 6H and Supplementary Fig. S7D), including an oncogenic transcription factor Mitf (Supplementary Fig. S7D). Taken together, these findings suggest that, while anti-PD-1 treatment reduced the percentage of the most dominant subcluster “Spp1 + ” in B16F10/pLKO tumors, it increased it in B16F10/shDRG2 tumors.

### Single-cell profiling of T cells within B16F10 tumors after anti-PD-1 treatment

**Fig. 7:**
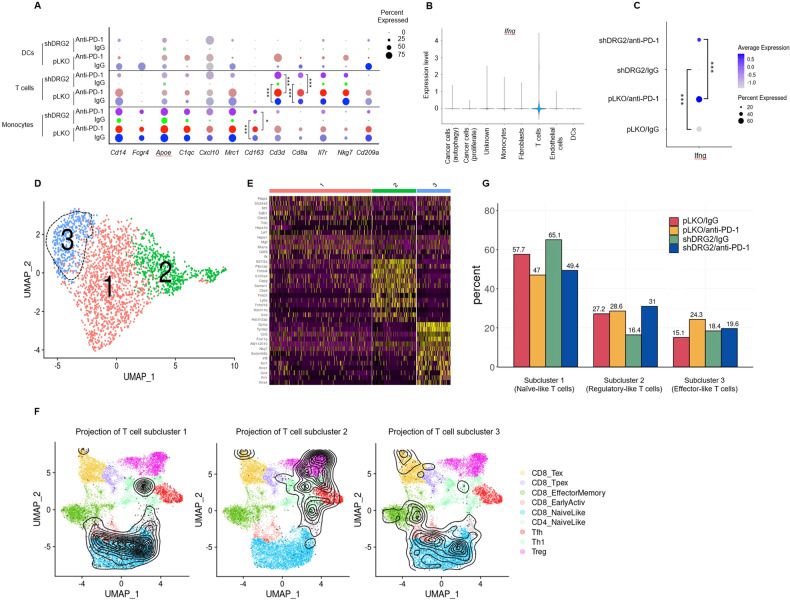
Anti-PD-1 immunotherapy fails to activate T cells in DRG2-depleted melanoma tumors. **A** A dot plot showing the percentage of cells expressing immune-related marker genes across the immune cell clusters in B16F10/pLKO or B16F10/shDRG2 tumors treated with negative control (IgG) or anti-PD-1. Student’s *t* test. ***P < 0.001. **B** A violin plot showing the log10 expression of IFN-γ gene across 8 clusters. **C** A dot plot showing both the average expression IFN-γ and the percentage of cells expressing IFN-γ in B16F10/pLKO or B16F10/shDRG2 tumors treated with negative control (IgG) or anti-PD-1. A darker color indicates higher average gene expression from the cells in which the gene was detected, and larger dot diameter indicates that the gene was detected in greater proportion of cells from the cluster. Student’s *t* test. ***P < 0.001. **D** UMAP of 2341 T cells showing 3 subclusters of T cells indicated by the color-coded legend. **E** A heatmap based on relative expression of the top-10 most differentially expressed genes for each T cell subcluster. **F** Annotation of T cell subclusters into reference atlas using ProjecTILs. The cells in the colored background refer to the cell types in the reference atlas, and the cells in the black circle represent the cells in the T cell subcluster 1, subcluster 2, and subcluster 3. Subcluster-defining genes are shown in Supplementary Fig. S8. **G** Relative contribution of each T cell subcluster (%) in B16F10/pLKO or B16F10/shDRG2 tumors treated with negative control (IgG) or anti-PD-1.

We next evaluated the effect of DRG2 depletion on transcriptional programming of TIICs: monocytes, T cells, and DC cells clusters. TIICs in DRG2-depleted tumors had significantly lower expression of genes associated with immune response such as *Cd14, Fcgr4, Apoe*, *C1qc*, *Cxcl10*, *Mrc1*, *Cd163*, *Cd3d*, *Cd8a*, *Il7r*, *Nkg7*, and *Cd209a* (Fig. 7A). A strong intra-tumor IFN-γ signature is a critical factor in determining the success of immunotherapy [58–60], and T cells are well-known key producers of IFN-γ [61]. Among 8 clusters, IFN-γ expression was mainly observed in the T cell cluster (Fig. 7B). Anti-PD-1 treatment elevated the percent of IFN-γ expressing T cells within both wild-type and DRG2-depleted tumors (Fig. 7C). However, the percent of IFN-γ expressing T cells were significantly lower in DRG2-depleted tumors than wild-type tumors treated with either IgG or anti-PD-1 antibody (Fig. 7C). These results suggest that DRG2 depletion in cancer cells attenuates the effect of anti-PD-1 antibody on the activation of tumor-infiltrating T cells. We next determined whether DRG2 depletion in cancer cells affects the expression of genes involved in the T-cell immune checkpoint. The frequency of T cells expressing genes involved in the T-cell immune checkpoint (*Pdcd1*, *Lag3*, and *Tigit*) was similar between wild-type B16F10 and B16F10/shDRG2 tumors treated with either IgG or anti-PD-1 antibodies (Supplementary Fig. S8A).

Tumor-infiltrating T cells could be divided into 3 subclusters (Fig. 7D). The T cells in subcluster 1 were heterogeneous in their expression of immune-related genes (Fig. 7E). However, T cells in subclusters 2 and 3 expressed specific gene sets involved in T cell function. T cells in subcluster 2 were characterized by high levels of genes associated with regulatory T cell phenotypes including *Ctla4*, *Capg*, *Tnfrsf4*, *Tnfrsf18* [62] and Foxp3 [63] (Fig. 7E and Supplementary Fig. S8B). In contrast, T cells in subcluster 3 had high expression of genes associated with effector function (*Gzma*, *Nkg7*, *Irf8*, *Orf1*, *Klra4*, *Prf1*) (Fig. 7E and Supplementary Fig. S8C). Projecting data of three T cell subclusters onto a reference atlas using ProjecTILs [64] revealed that T cells in subcluster 1, 2, and 3 were mostly projected to the naive-like, regulatory-like, and effector-like T cells, respectively (Fig. 7F).

We next compared the changes in the abundance of three T cell subclusters between wild-type and DRG2-depleted tumors after anti-PD-1 treatment. T cell populations in subcluster 1 decreased in both wild-type and DRG2-depleted tumors after anti-PD-1 treatment (Fig. 7G). However, T cell populations of subcluster 2 and 3 in DRG2-depleted tumors showed distinct patterns from those in wild-type tumors. After anti-PD-1 antibody treatment, the effector-like T cell population of subcluster 3 was expanded in wild-type tumors but not in DRG2-depleted tumors (Fig. 7G). In contrast, the regulatory-like T cell population of subcluster 2 was expanded in DRG2-depleted tumors but not in wild-type tumors after anti-PD-1 treatment (Fig. 7G). Collectively, these results suggest that, while anti-PD-1 treatment expands the effector-like T cell population (subcluster 3) in DRG2-expressing tumors, it does not in DRG2-depleted tumors. Instead, anti-PD-1 treatment expands the regulatory-like T cell population (subcluster 2) in DRG2-depleted tumors.

## Discussion

Clinical studies revealed that the success of PD1–PD-L1 blockade in melanoma correlates with PD-L1 expression levels in tumor cells [65, 66]. However, the outcome of PD1–PD-L1 blockade therapy is not always in accordance with PD-L1 expression in melanoma [9, 15, 16], suggesting that PD-L1 expression alone is a poor predictor for response to PD1–PD-L1 blockade therapy. Hence, a deeper understanding of the PD-L1 blockade is required to improve the clinical response rate and efficacy of PD1–PD-L1 blockade in melanoma patients with positive PD-L1 expression. Here, we have shown that even though DRG2-depleted cancer cells express high level of PD-L1, mice bearing DRG2-depleted melanoma did not respond to anti-PD-1 treatment. In addition, from data analysis of a melanoma patient cohort under anti-PD-1 treatment [52], we found that low DRG2 protein expression correlated with a poor clinical response of melanoma patients to anti-PD-1 therapy.

There are many tumor intrinsic factors which can induce resistance to ICBs [67]. For example, activation of the epithelial-mesenchymal transition (EMT) program promotes an immune suppressive microenvironment and resistance to ICBs [45–47]. Reduced expression of MHC class I molecules and PD-L1 in cancer cells confers resistance to ICBs [67–69]. However, our study revealed that DRG2-depleted tumor cells neither induced expression of genes involved in EMT nor suppressed the expression of PD-L1 and MHCs both before and after IFN-γ treatment. In contrast, even though we did not determine the detailed mechanism, DRG2 depletion enhanced STAT1/IRF1 signaling pathway and the expression of PD-L1 in cancer cells after IFN-γ treatment. In addition, single-cell RNA-seq analysis of tumor cells within B16F10 tumors revealed that DRG2 depletion did not generate a specific cluster of tumor cells expressing transcriptional programs involved in the resistance to PD-1 blockade.

How do DRG2-depleted tumor cells escape anti-PD-1 antibody-mediated inhibition without inducing well-known transcriptional programs which contribute to ICB resistance? Anti-PD-1 ICB inhibits tumor growth through reinvigorating anti-tumor immune response mainly mediated by T cells [48, 70]. In this study, we provided evidence suggesting that anti-PD-1 ICB fails to reinvigorate T cells in DRG2-depleted tumors. First, while anti-PD-1 treatment expanded effector-like T cell population in wild-type tumors, it did not do so in DRG2-depleted tumors. Second, after anti-PD-1 treatment, the percent of IFN-γ-expressing cells in the DRG2-depleted tumors was lower than that from wild-type tumors. Third, while tumor cell population in the most dominant cluster (cluster Spp1 + ) decreased after anti-PD-1 treatment in wild-type tumors, the same population expanded in DRG2-depleted tumors after anti-PD-1 treatment. These results suggest that anti-PD-1 antibodies fail to reinvigorate effector-like T cells in DRG2-depleted tumors, which might lead to failure in inhibiting tumor growth.

The next question was how DRG2 depletion in cancer cells inhibits the efficacy of anti-PD-1 antibody in reinvigorating effector-like T cells. PD-1/PD-L1 blockade by anti-PD-1 antibody is theoretically efficient for PD-L1 on surface membranes [32, 34, 35]. Many membrane proteins are shuttled between the recycling endosomes and cell surface [22, 23]. A large proportion of membrane PD-L1 also continuously undergoes internalization and recycling of PD-L1 to maintain the level at the membrane surface [33, 34]. Deactivation of Rab5 on early endosomes is required for endosome trafficking from early endosomes to recycling endosomes [30, 31]. Previously, we reported that DRG2 depletion leads to defects in deactivation of Rab5 on early endosomes and delays endosome recycling, which results in prolonged-localization of surface receptors in early endosomes [37, 38]. Thus it is predicted that DRG2-depleted cells would show defects in recycling of endosomal PD-L1. In this study, we found that DRG2 depletion delayed the trafficking of PD-L1 from Rab5 early endosome to Rab11 recycling endosome in cancer cells, which led to a decrease in the surface membrane PD-L1. As supportive evidence, we found that PD-L1 in DRG2-depleted cancer cells showed reduced-binding to recombinant PD-1 and defects in inhibiting T cell activity. A recent study revealed that CMTM6 regulates recycling of PD-L1 in cancer cells and CMTM6 depletion reroutes the internalized PD-L1 to lysosomal degradation of PD-L1, which leads to a decrease in PD-L1 level in cancer cells [34]. Unlike CMTM6, DRG2 depletion prolonged the localization of PD-L1 at Rab5 early endosomes and increased the total PD-L1 level in cancer cells. Our results suggest that even though DRG2 depletion enhanced the PD-L1 level in cancer cells, it inhibits the endosomal trafficking of PD-L1 and decreases the PD-L1 level on the surface of cancer cells, which results in poor response to anti-PD-1 therapy.

Here, we demonstrate that DRG2 is a key regulator of endosomal trafficking of PD-L1 in cancer cells, and DRG2 depletion inhibits the recycling of PD-L1, resulting in accumulation of PD-L1 in endosomes. Since PD-1/PD-L1 blockades are effective on membrane surface PD-L1 [32, 34, 35], the endosomal accumulation of PD-L1 in DRG2-depleted cells limited the efficacy of anti-PD-1 therapies. Overall, our results suggest that DRG2 modulates anti-PD-1 therapy through regulation of endosomal trafficking of PD-L1 in cancer cells. Currently, PD-L1 expression by immunohistochemistry (IHC) on tumor cells is widely used for predicting response to PD-1/PD-L1 blockade therapy in cancer patients [9, 71]. Considering that currently used IHC tests detect both surface membranes and intracellular PD-L1, DRG2-depleted tumors with the endosomal accumulation of PD-L1 may test positive by IHC but may not respond to PD-1/PD-L1 blockades. This study can provide insight into how to increase the correlation of PD-L1 IHC with the clinical outcomes to PD-1/PD-L1 blockades. Follow-up studies are needed to determine correlation between DRG2 expression and response to PD-1/PD-L1 blockades among patients with PD-L1 IHC positive and to further explore whether combining DRG2 with PD-L1 test improves patient selection for PD-1/PD-L1 blockade therapy.

## Methods

### Cell culture

Murine melanoma B16F10 (CRL-6475, KCLB 80008), murine colorectal carcinoma CT26 (CRL-2638, KCLB 80009), and human melanoma SK-MEL-28 (HTB-72, KCLB 30072) cell lines were purchased from the Korean Cell Line Bank (KCLB-Seoul, Korea). Cells were cultured in DMEM media and supplemented with 10% fetal bovine serum (WELGENE, Korea) at 37 °C in a humidified atmosphere of 5% CO_2_. To induce PD-L1, B16F10 and SK-MEL-28 cells were incubated for 24 h with the indicated concentration of recombinant mouse IFN-γ (PeproTech, 315-05) and 10 ng/ml recombinant human IFN-γ (PeproTech, 300-02), respectively.

### Analysis of the GEPIA database

GEPIA (http://gepia.cancer-pku.cn/) is a web tool for cancer and normal gene-expression profiling and interactive analyses based on TCGA and Genotype-Tissue Expression (GTEx) data [72]. To determine the effect of DRG2 expression on the correlation between PD-L1 expression and survival, patient samples were divided into two groups (the high- and low-expression group) according to the median expression level of DRG2. Each group was analyzed for the correlation between PD-L1 expression and overall survival (OS) using GEPIA. Log-rank *P* < 0.05 was considered statistically significant.

### Integrative analysis of bulk transcriptomic and proteomic data from anti-PD-1-treated melanoma patient cohorts

We collected the anti-PD1 treated melanoma patient cohorts with available RNA-seq (GEO, GSE78220) or proteomic data (https://www.sciencedirect.com/science/article/pii/S0092867419309006) [49]. We adopted Salmon v1.4.0 to obtain read counts and TPMs for each dataset using the default parameter setting. We computed expression-response associations in anti-PD-1-treated melanoma patients and then examined the concordance of the two associations to evaluate the potential of DRG2 for anti-PD-1 therapy in melanoma. We adopted a log-rank test (by ggsurvplot with the option log.rank.weights = “n” to identify early survival differences) for survival analysis. The patients in a cohort were divided into high- and low-DRG2 expression groups based on the median expression. We then computed the association between the overall survival rate and the expression groups.

### Plasmids, siRNAs, and transfections

Plasmid construct pLKO-DRG2-shRNA containing short hairpin RNA (shRNA) against mouse DRG2 (pLKO-mDRG2-shRNA, TRC0000047195) and human DRG2 (pLKO-hDRG2-shRNA, TRC0000047195), and non-target shRNA control vector (MISSION pLKO.1-puro non-mammalian shRNA control plasmid DNA, SHC002) were purchased from Sigma. Small interfering RNAs (siRNAs) against mouse/human siDRG2 (simDRG2/sihDRG2) and control siRNA (scRNA) were designed by Integrated Device Technology and were purchased from GenePharma.

To generate DRG2-depleted cells, B16F10/shDRG2 and SK-MEL-28/shDRG2, B16F10 and SK-MEL-28 cells were transfected with pLKO-mDRG2-shRNA and pLKO-hDRG2-shRNA, respectively, and selected by puromycin (Sigma P9620). Non-target shRNA in pLKO.1-puro was used to generate control cells, B16F10/pLKO and SK-MEL-28/pLKO. Cells were transfected with the various plasmid constructs using TurboFect (Thermo Scientific). To monitor transfection efficiency, the GFP expression vector pEGFP-N1/C1 (Clontech) was co-transfected with the plasmid construct. Cells were used for further study after confirming transfection efficiency (>80%).

### Murine in vivo experiments

Female C57BL/6 mice were purchased from Orient Bio (Busan, Korea). All mice were used between 8 and 12 weeks of age. Water and food were provided ad libitum. The mice were housed at the laboratory animal facility at Ulsan University for Life Sciences under specific pathogen-free conditions and all animal procedures were approved by the Institutional Animal Care and Use Committee of the Meta-inflammation Research Center (permit number JWP-21-020). For in vivo experiments, B16F10/pLKO and B16F10/shDRG2 cells (5 × 10^5^ cells/mouse in 100 μL) were resuspended in FBS-free media and were s.c. injected in the upper right dorsal flank of 8-week-old female C57BL/6 mice (Oriental Bio, Busan, Korea). For in vivo therapeutic studies, mice were randomized into two groups with *n* = 11 mice/group based on tumor volume when tumor volume reached ~100 mm^3^. Five doses of 10 mg/kg anti-PD-1 (Novartis, clone 1D2) or isotype control (Novartis, clone MOPC-21) were administered intraperitoneally (i.p.) once every 5 days. The tumor volume was measured 2–3 times per week (length × width) with a digital caliper. The tumor volume was determined using the formula ([l × w2] × 3.14159)/6. Survival endpoint was defined as either tumor volume exceeding 1000 mm^3^ or if the tumors became necrotic, and mice were humanely euthanized.

### Real-time and semiquantitative RT-PCR

Total RNA from cells was extracted using TRIzol Reagent (Invitrogen). After measuring the RNA concentration on a NanoDrop™ 2000 system (Thermo Fisher Scientific, Waltham, MA, USA), cDNA was synthesized using 2 μg of total RNA and M-MLV reverse transcriptase (Promega). cDNA was used as a template for quantitative real-time PCR (qRT-PCR) with gene specific primers listed in Supplementary Table S4. Real-time qRT-PCR was conducted using SYBR Green PCR Master Mix (QIAGEN) on an ABI 7500 Fast Real-Time PCR System (Applied Biosystems). Semiquantitative RT-PCR was performed using Taq polymerase (Solgent, Daejeon, Korea).

### Endo H treatment

Cells were incubated for 24 h with recombinant IFN-γ as described above. Cell lysates were treated with 1.000 unit endoglycosidase H (endo H) (Promega, V4871) for 17 h at 37 °C in the presence the buffers recommended by the supplier. Samples were loaded onto an SDS-polyacrylamide gel and analyzed by immunoblotting using the anti-PD-L1 antibody (R&D Systems, AF1019).

### Western botting

Proteins in cell extracts were separated by SDS-PAGE and were probed with appropriate dilutions of anti-mouse and human DRG2 (Proteintech, 14743-1-AP), mouse anti-PD-L1, human anti-PD-L1 (Cell signaling, 136845), anti-STAT1 (Santacruz, SC-464), anti-phospho STAT1 (cell signaling, 9177), anti-β-actin (Sigma, A5441). Immunoreactivity bands were detected using Pierce ECL Western blotting substrate (Thermo Scientific).

### Phenotypic analysis of tumor-infiltrating immune cells (TIICs)

Tumors were collected at 15 days after s.c. injection of B16F10 cells. The tumor masses were minced into 3–5 mm^2^ slices and dissociated by incubating in 1 mg/ml collagenase type IV (Sigma, C5138) and 20 mg/ml DNase (Sigma, D5025) for 30 min with rotation. The resulting mixture was suspended in FACS buffer (5% FBS in PBS) and passed through a 70-μm nylon cell strainer (SPL, 93070) to obtain a single-cell suspension. Fc receptors were blocked using TruStain FcX (Biolegend, 101319) according to the manufacturer’s protocol, and cells were stained with the following fluor-conjugated antibodies for 1 h on ice: PE anti-mouse CD8 (Invitrogen, 12-0081-82), PerCP anti-mouse CD3 (Invitrogen, 45-0031-82), PE anti-mouse NK1.1 (Invitrogen, 12-5941-82), PE anti-mouse CD11c (Invitrogen, 12-0114-82), FITC anti-mouse F4/80 (Invitrogen, 11-4801-82), and PE anti-CD206 antibodies (Biolegend, 141706). To detect Intracellular IFNγ, cells were fixed with 4% paraformaldehyde (Sigma, F8775) for 15 min and were permeabilized with 0.2% Triton X-100 (Sigma, T9284) for 10 min and were probed with FITC anti-mouse IFNγ (Biolegend, 505805). Data were acquired on a FACSCantoII (BD Biosciences) and were analyzed using FACSDiva software.

### Flow cytometry analysis for PD-1 and PD-L1 binding

After stimulation for 24 h with recombinant IFN-γ described above, cells were suspended using trypsin-EDTA (Welgene, LS-015-01) and incubated with recombinant PD-1 human Fc (1 μg/ml) (R&D Systems, 1021-PD) for 10 min and 30 min. After washing with ice-cold PBS buffer containing 0.5% BSA and 0.02% NaN_3_), cells were incubated with Alexa Fluor 488-conjugated anti-human IgG Fc antibody (Invitrogen, A11013) for 30 min. Cells were washed and fixed with 4% paraformaldehyde in PBS and analyzed using FACSCantoII (BD Biosciences).

### Confocal microscopy

After stimulation for 24 h with recombinant IFN-γ as described above, cells were washed three times with ice-cold phosphate-buffered saline (PBS) and fixed with 4% paraformaldehyde (18814-10; Polysciences). Endogenous PD-L1 was stained using anti-mouse PD-L1 antibody conjugated with Alexa Fluor 647 (R&D System, FAB9078R) and anti-human PD-L1 antibody conjugated with Alexa Fluor 647 (R&D System, FAB1562R). Endogenous DRG2 was stained using anti-mouse and human DRG2 (Proteintech, 14743-1-AP). Early endosome marker Rab5 and recycling endosome marker Rab11 were stained using anti-mouse Rab5 antibody (BD Transduction Laboratories, 610725), and anti-mouse Rab11 antibody (BD Transduction Laboratories, 610656), respectively. *Triticum vulgare* lectin (wheat germ lectin) conjugated with FITC (EY Laboratories, F-2101-5) was used to stain cytoplasmic membranes. Nuclei were stained with 4′,6-diamidino-2-phenylindole (DAPI, Life Technologies). After mounting, the cells were visualized using a Nikon A1 plus at the University of Wisconsin-Madison, Optical Core at Biochemistry USA. All images were saved as TIFF files, and their contrast was adjusted with ImageJ (version 1.19 m; National Institutes of Health, Bethesda, MD). Object based colocalization analysis was performed using MatCol [73]. Images were analyzed with MetaMorph 7 software (Universal Imaging) and Imaris Bitplane-9 at the University of Wisconsin, Madison, WI, USA.

### Ratiometric FRET microscopy to detect Rab5 activity

B16F10 cells were cultured in DMEM containing recombinant mouse IFN-γ Protein 500 I.U. (R & D systems, 485-MI) for 24 h. Cells were incubated at 37 °C for the indicated time. We used pRaichu-Rab5 as FRET probes for Rab5 and Rac1 GTPases, respectively. Raichu-Rab5 consists of Venus, the amino-terminal Rab5-binding domain of EEA1 (amino acid residues 36–218), SECFP, and Rab5a [74]. FRET analysis for Rac1 and Rab5 was conducted as described previously [75]. Briefly, control and DRG2-depleted B16F10 cells were transfected with pRaichu-Rab5. Images were captured using a Nikon A1R-Si+ Confocal Microscope. CFP and YFP images were processed using MetaMorph software (Universal Imaging, West Chester, PA), USA. After background subtraction, FRET ratio images were generated using MetaMorph software and were visualized in intensity-modulated display mode. In this display mode, eight colors from red to blue were used to represent the FRET ratio (YFP/CFP), with the intensity of each color indicating the mean intensity of YFP and CFP.

### Mouse CD4^+^ T cell co-culture and IL-2 assays

Untouched mouse CD4^+^ T cells were isolated from mouse spleens using a MACS column in the CD4 + T cell Isolation Kit (#130-095-248, Miltenyi Biotech). They were activated with anti-CD3 (BD Biosciences, 553057) and anti-CD28 (BD Biosciences, 553294) according to the manufacturer’s protocols. B16F10-pLKO and B16F10-shDRG2 cells were treated with IFNγ for 24 h. To analyze the effect of cancer cells on T cell activity, IFNγ-treated B16F10 cells and activated CD4^+^ T cells were co-cultured in a 24-well plate (SPL, 3002) at a ratio of 1:5 (B16F10: CD4^+^ T) for 48 h. We used a co-culture insert system to confirm the paracrine effect of cancer cells on T cell activity. IFNγ-treated B16F10 cells (5 × 10^3^ cells/well) were placed on the upper layer of a cell culture insert, and activated CD4^+^ T cells (2.5 × 10^4^ cells/well) were added to the bottom layer of the well plate (Costar, 3422 transwell) and incubated for 48 h. The secreted IL-2 level in the medium was measured using mouse IL-2 ELISA Kits (Biolegend, 431005) according to the manufacturer’s protocol.

### Bulk RNA-Seq from IFN-γ-treated B16F10 cells

We performed RNA-Seq on total RNA samples (RIN above 8.5) from B16F10-pLKO and B16F10-shDRG2 cells stimulated with 5 ng/ml recombinant mouse IFN-γ (PeproTech, 315-05) for 24 h. The cDNA library was prepared with 1.0 μg of total RNA following the manufacturer’s recommendations of the TrueSeq RNA library Preparation Kit (Illumina, San Diego, CA, USA), followed by paired-end sequencing (2 × 100 bp) using the HiSeq1500 platform (Illumina, San Diego, CA, USA). cDNAs were amplified according to the RNAseq protocol provided by Illumina and sequenced using an Illumina HiSeq 2500 system to obtain 150-bp paired-end reads. The sequencing depth for each sample was >20 million reads. The raw sequencing reads were filtered and trimmed using Trim Galore0.6.7. The filtered reads were aligned to the reference genome mm10 using Spliced Transcript Alignment to a Reference (STAR) software tool version 2.6.1d [76] with the parameter 2pass. Transcript abundance was estimated using the quasi-mapping method, and gene-level counts were generated using Salmon version 1.5.2 [77]. Hierarchical clustering was performed to display DEGs pattern using complete linkage and Euclidean distance as a measure of similarity, which, along with other RNA-Seq data visualizations, was performed using R packages (www.r-project.org). Statistical significance for the DEGs was set to *P* < 0.05 and *q* < 0.05. A volcano plot of DEGs was created using DEseq2 [78]. A heatmap based on significantly changed DEGs was generated using heatmap.2. Pathway analysis of bulk RNA-Seq was performed using the DESeq2 results that were run through the R package ClusterProfiler using the Hallmark GeneSets from MSigDB (Broad Institute) [79]. Plots of running enrichment score were generated with R.

### scRNA-Seq of tumors treated with anti-PD-1 antibody in vivo

#### Sample collection and processing

B16F10/pLKO and B16F10/shDRG2 tumors were collected 3 days after the 2nd administration of anti-PD-1 antibody or isotype control IgG. The tumor samples were first mechanically dissociated using a scalpel, then enzymatically dissociated in digestion medium (2 mg/ml Collagenase P (Sigma Aldrich) and 0.2 mg/ml DNAse I (Roche) in DMEM (Thermo Fisher Scientific)). Red blood cells were removed from the cell suspension using red blood cell lysis buffer (Roche), and the cells were filtered using a 40-µm Flowmi tip strainer (VWR). The number of living cells was determined using a LUNA automated cell counter (Logos Biosystems).

#### Single-cell RNA-sequencing

We performed single-cell RNA-seq on the single-cell suspension using the Chromium Single Cell 3’ Solution from 10x Genomics according to the manufacturer’s instructions. Up to 5000 cells were loaded onto a 10x Genomics cartridge for each sample. Cell-barcoded 5′ gene expression libraries were sequenced on an Illumina NextSeq and/or NovaSeq6000 system.

#### Single-cell data pre-processing and quality control

Cell Ranger v3.1.0 was used to demultiplex the FASTQ reads, align them to the mm10 mouse transcriptome, and extract their cell and unique molecular identifier (UMI) barcodes. The output of this pipeline is a digital gene expression (DGE) matrix for each sample, which records the number of UMIs for each gene that are associated with each cell barcode. Raw gene expression matrices were analyzed using the Seurat v3 R package [50, 80]. As a quality control step, we first filtered out cells with fewer than 100 genes and genes expressed in less than 0.1% of cells using zero as a cut-off for UMI counts. We further removed cells based on mitochondrial gene content, UMI counts, and gene counts (mitochondrial% counts ≥10%, UMI counts >9000, gene counts >2500). The filtered gene-expression matrix (14,609 genes and 62,956 cells: B16F10/pLKO + IgG, 13,825 cells; B16F10/pLKO + anti-PD-1, 14,466 cells; B16F10/shDRG2 + IgG, 18,785 cells; B16F10/shDRG2 + anti-PD-1, 15,880 cells) was normalized using the NormalizeData function with the LogNormalize normalization method and scale factor equal to 10,000.

### scRNA-seq clustering leading to cell types

To remove the batch effect for the four samples, we conducted anchor-based CCA integration workflow implemented in Seurat v3. On the integrated expression space, 2000 highly variable genes were identified, and principal component analysis (PCA) was applied to the dataset to reduce dimensionality after regressing for the number of UMIs (counts), percentage mitochondrial genes and cell cycle (S and G2M scores were calculated by the CellCycleScoring function in Seurat). The 20 most informative principal components (PCs) were used for the K-nearest neighbor (KNN) graph construction followed by clustering and Uniform Manifold Approximation and Projection for dimension reduction (UMAP) [81, 82]. Clusters in the resulting two-dimensional UMAP representation consisted of distinct cell types, which were identified based on the expression of marker genes. DEGs that functionally characterized the clusters were defined by the Wilcoxon Rank Sum test implemented in the FindAllMarkers function from Seurat. We used Enricher [83] web-based software to identify enriched KEGG pathway terms for the marker genes. ProjecTILs [64] was used to project the scRNAseq data of T cells into reference T cells.

### Pseudo-bulk analysis of scRNA-seq

To compare gene expression between cancer cells of four samples, we performed pseudo-bulk analysis based on the clustering result. The summed UMIs of each gene for all the cancer cells in each sample were considered as mRNA counts of bulk RNA-seq results of cancer cells. We calculated the *P* values in Supplementary Fig. S7 using EdgeR [84].

### Statistical analysis

Differences in the expression of genes were evaluated using the Student’s *t* test or one-way ANOVA. A *P* value less than 0.05 was considered statistically significant.

### Supplementary information

Supplemental Material

Table S1

Table S2

Table S3

Table S4

Original data files

## Data Availability

The bulk RNAseq of B16F10 cells with DRG2 depletion (GSE231972) and scRNAseq B16F10 tumors with DRG2 depletion (GSE231973) can be downloaded from the Gene Expression Omnibus database.
