# Supplementary material for: DRG2 is required for surface localization of PD-L1 and the efficacy of anti-PD-1 therapy
Source: Cell Death Discov. 2024 May 27;10:260. doi: 10.1038/s41420-024-02027-x (PMC11130180; doi:10.1038/s41420-024-02027-x)
Supplement: Supplementary file 1 — Supplemental Material [file 41420_2024_2027_MOESM1_ESM.docx]

**DRG2 is required for surface localization of PD-L1 and the efficacy of anti-PD-1 therapy**

Seong Hee Choi^1,2^, Muralidharan Mani^3^, Jeonghwan Kim^4^, Wha Ja Cho^1^, Thomas FJ Martin^3^, Jee Hyun Kim^2^, Hun Su Chu^2^, Won Jin Jeong^2^, Young-Wook Won^2,5^, Byung Ju Lee^1,6^, Byungyong Ahn^6,7^, Junil Kim^4^, Do Yong Jeon^1^, Jeong Woo Park^1,6^

^1^Department of Biological Sciences, University of Ulsan, Ulsan 44610, Korea

^2^RopheLBio, B102, Seoul Forest M Tower, Seoul 04778, Korea

^3^Department of Biochemistry, University of Wisconsin-Madison, Madison, WI 53706-1544, USA

^4^School of System Biomedical Science, Soongsil University, Seoul 06978, Korea

^5^Department of Biomedical Engineering, University of North Texas, Texas 76203-5017, USA

^6^Basic-Clinic Convergence Research Institute, University of Ulsan, Ulsan 44610, Korea

^7^Department of Food Science and Nutrition, University of Ulsan, Ulsan 44610, Korea

**Running title**: DRG2 regulates endosomal trafficking of PD-L1.

**Correspondence**

Jeong Woo Park, [jwpark@ulsan.ac.kr](mailto:jwpark@ulsan.ac.kr); Do Yong Jeon, [dyjeon@empas.com](mailto:dyjeon@empas.com); Junil Kim,

Seong Hee Choi, Muralidharan Mani, and Jeonghwan Kim equally contributed.


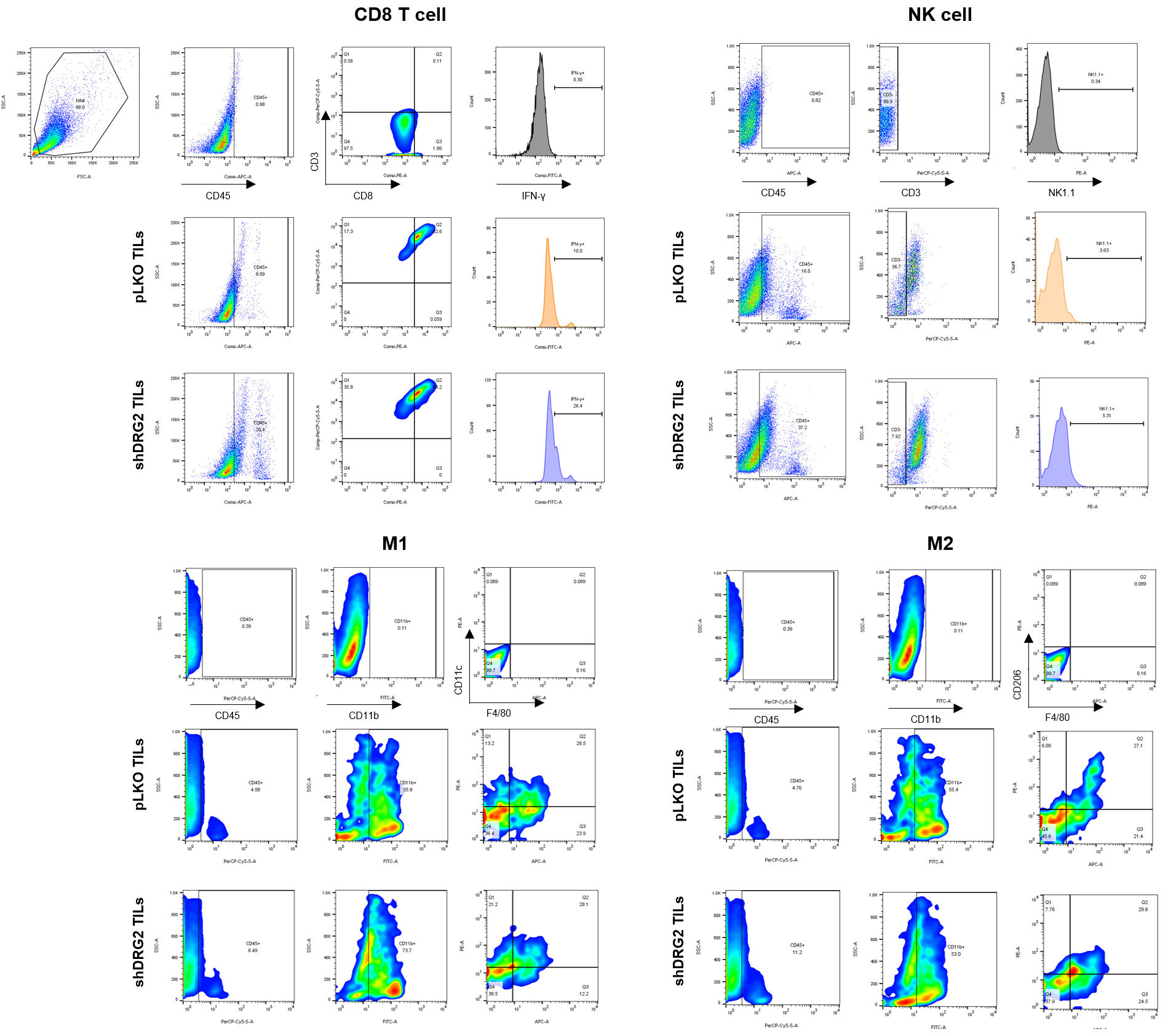


**Supplementary Fig. S1 DRG2 deficiency in B16F10 cells increases percentage of CD8^+^IFNγ^+^T cells within the TIICs. Related to Fig. 1.** Tumors were collected at 15 days after s.c. injection of B16F10 cells. TIICs were FACS analyzed for CD8^+^IFNγ^+^ T cells, CD3^+^NK1.1^+^ NK cells, CD11C^+^F4/80^+^ M1, and CD206^+^F4/80^+^ M2 cells. Representative FACS plots and numbers in quadrants indicate the percentages of positive cells in that area.


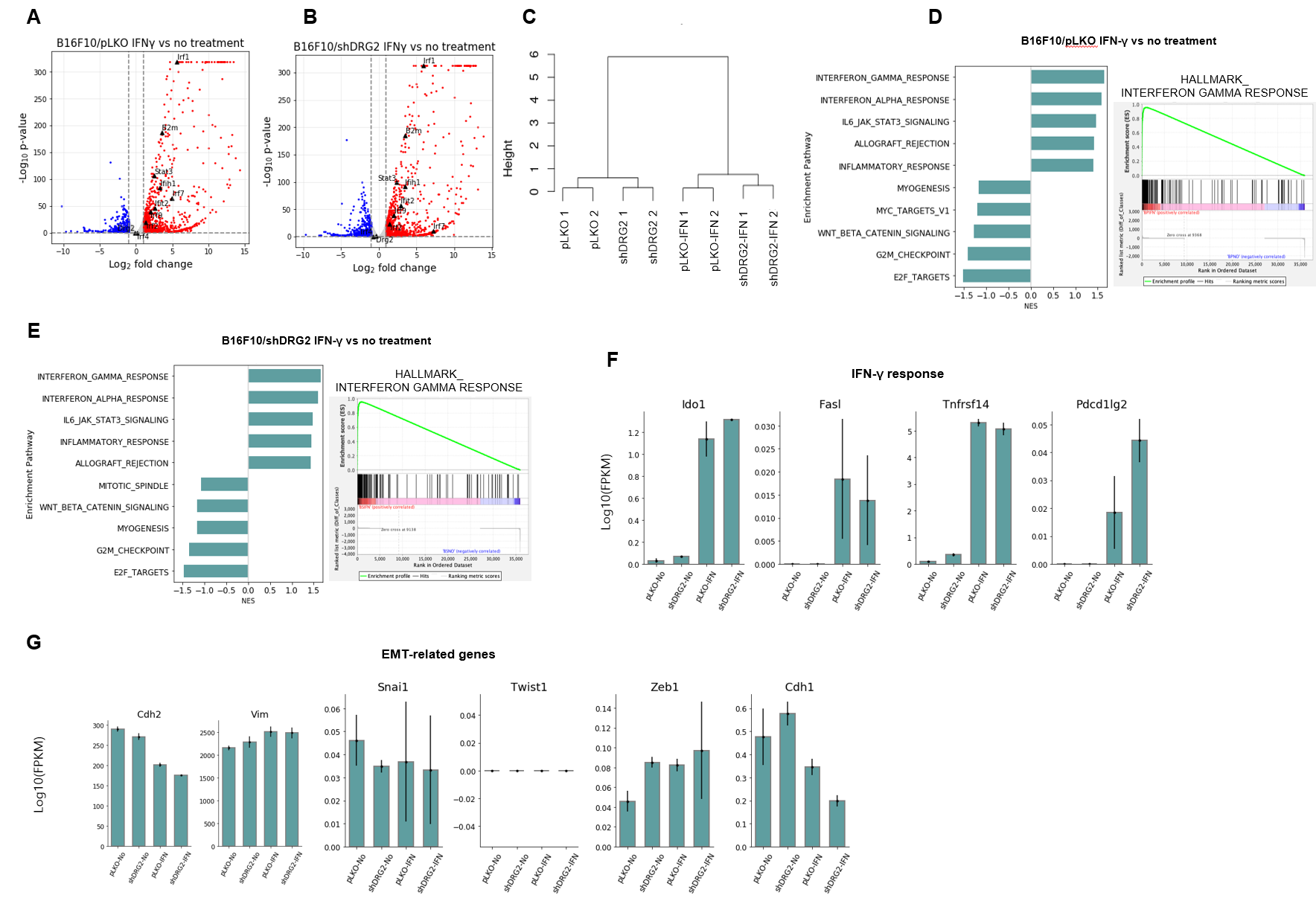


**Supplementary Fig. S2 RNA-Seq analysis of B16F10/pLKO and B16F10/shDRG2 cells after treatment with IFNγ**. **Related to Fig. 2**. B16F10/pLKO and B16F10/shDRG2 cells were treated with 5 ng/ml IFNγ for 24 h and their transcriptome profiles were analyzed by RNA-Seq. **A,B** Volcano plots of differentially expressed genes (DEGs) in IFNγ-treated B16F10 compared with non-treated B16F10 cells. The y-axis corresponds to the significance level represented with the -log_10_P value, and the x-axis displays the log_2_ (FC) value. Blue and red dots represent significant (adj.*p* < 0.05 and |Log2FC| ≥ 1.5) DEGs in (**A**) IFNγ-treated B16F10/pLKO and (**B**) IFNγ-treated B16F10/shDRG2 cells. Dotted horizontal line indicates an adj.*p* = 0.05, and dotted vertical lines indicate a mean |log2FC| of 1.5. **C** Unsupervised hierarchical clustering of RNA-Seq data from non-treated and IFNγ-treated B16F10/pLKO and B16F10/shDRG2 cells. **D,E** GSEA. (**D**) Bar plot of enriched GSEA pathways in IFNγ-treated B16F10/pLKO cells vs. non-treated B16F10/pLKO cells. Enrichment plot of IFNγ responses positively enriched in IFNγ-treated B16F10/pLKO. (**E**) Bar plot of enriched GSEA pathways in IFNγ-treated B16F10/shDRG2 cells vs. non-treated B16F10/shDRG2 cells. Enrichment plot of IFNγ responses positively enriched in IFNγ-treated B16F10/shDRG2 cells. **F,G** Expression of genes involved in (**F**) IFNγ response and (**G**) epithelial-mesenchymal transition (EMT) in non-treated and IFNγ-treated B16F10/pLKO and B16F10/shDRG2 cells. The y-axis corresponds to fragments per kilobase of exon model per million mapped reads (FPKM) measured by RNA-Seq (EdgeR at FDR < 0.05).


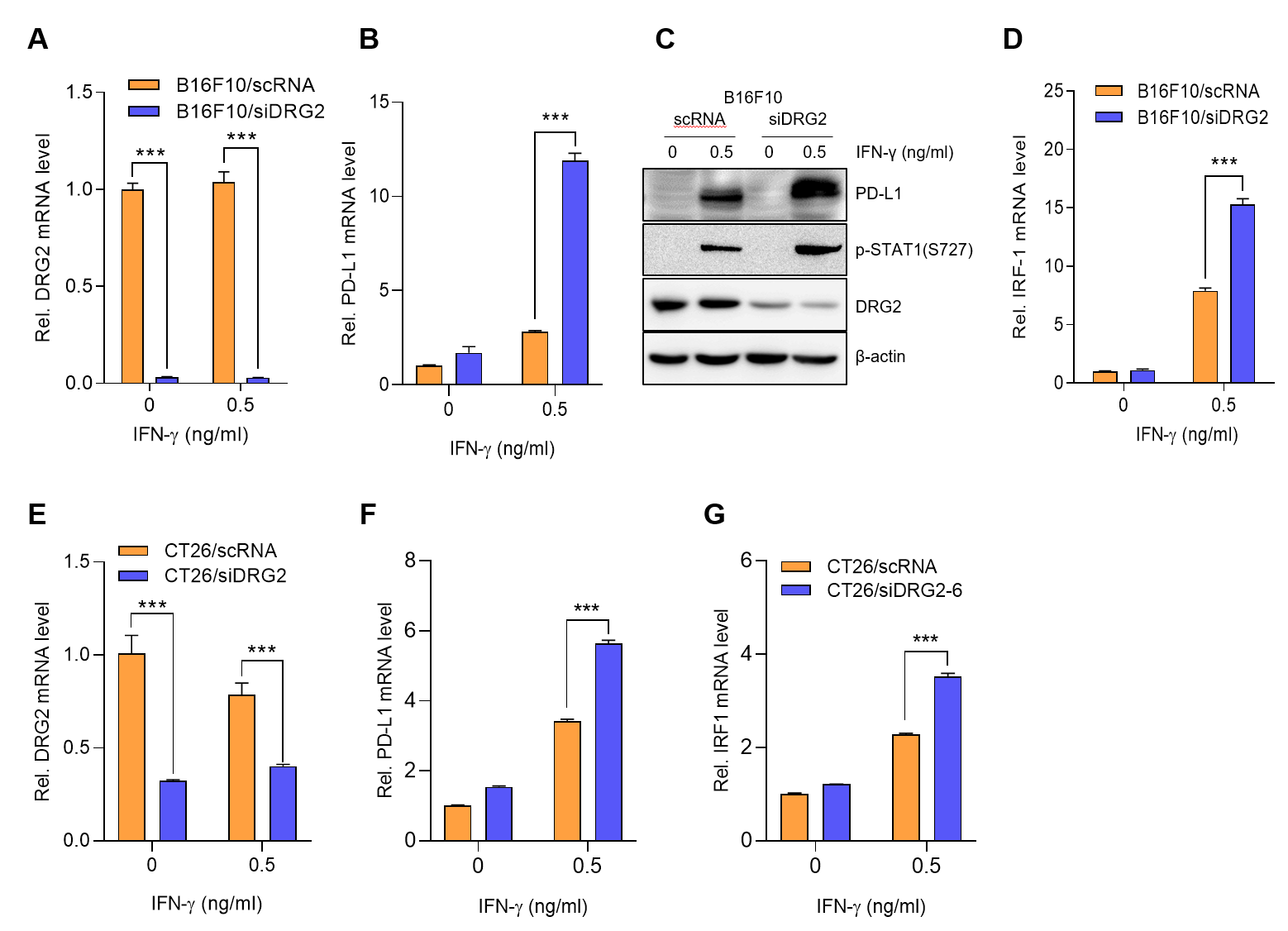


**Supplementary Fig. S3 DRG2 deficiency enhances IFN-γ responses and PD-L1 expression in cancer cells. Related to Fig. 2. A-D** B16F10/scRNA and B16F10/siDRG2 cells were treated with 0.5 ng/ml IFNγ for 24 h. (**A**) qRT-PCR analysis for *DRG2* expression. (**B**) qRT-PCR analysis for *PD-L1*. (**C**) Western blot analysis for PD-L1 and phosphorylated STAT1. (**D**) qRT-PCR analysis for *IRF1*. **E-G** CT26/scRNA and CT26/siDRG2 murine colon cancer cells were treated with 0.5 ng/ml IFNγ for 24 h. (**E**) qRT-PCR analysis for *DRG2*. (**F**) qRT-PCR analysis for PD-L1. (**G**) qRT-PCR analysis for *IRF1*. Values are the mean ± SD of two independent experiments (n = 3 per group per experiment). Student’s t-test. ****p*<0.001. ns, not significant.


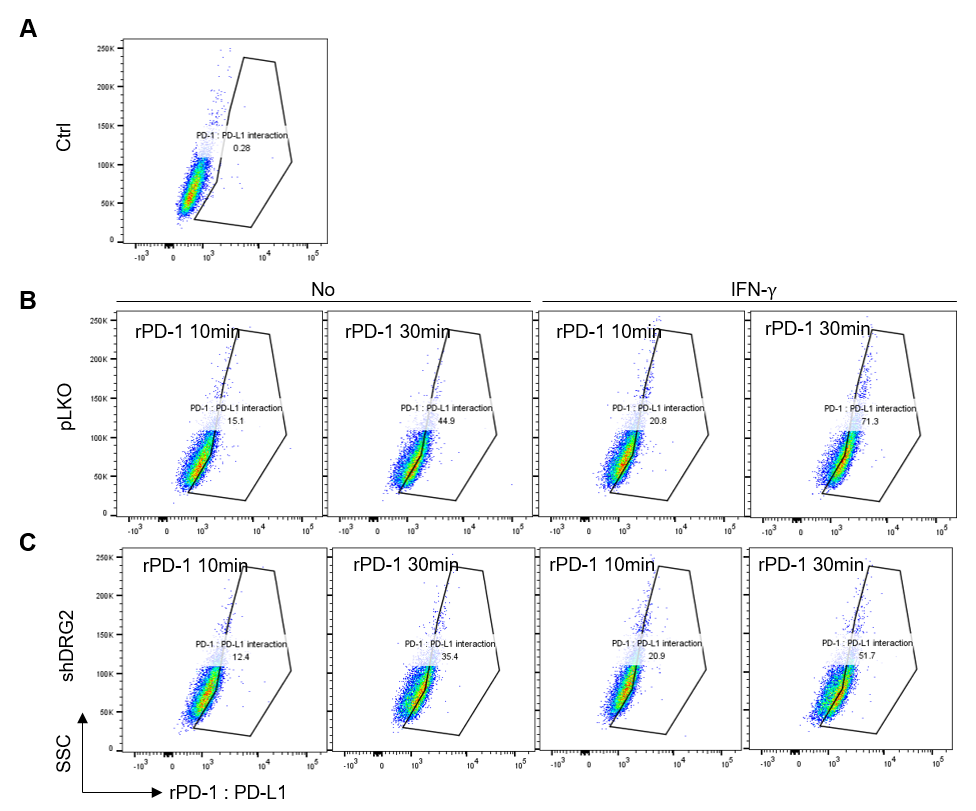


**Supplementary Fig. S4 PD-L1 in DRG2-depleted B16F10 cells shows defect in binding with recombinant PD-1 protein. Related to Fig. 3.** IFN-γ-treated B16F10/pLKO and B16F10/shDRG2 cells were incubated with Fc-conjugated recombinant PD-1 for 10 min and 30 min. Cells were FACS analyzed for cell-bound PD-1 after staining with Alexa Fluor 488-conjugated anti-human IgG Fc antibody. Representative FACS plots obtained from **A** non-treated B16F10/pLKO cells, **B** recombinant PD-1-treated B16F10/pLKO cells, and **C** recombinant PD-1-treated B16F10/shDRG2 cells.


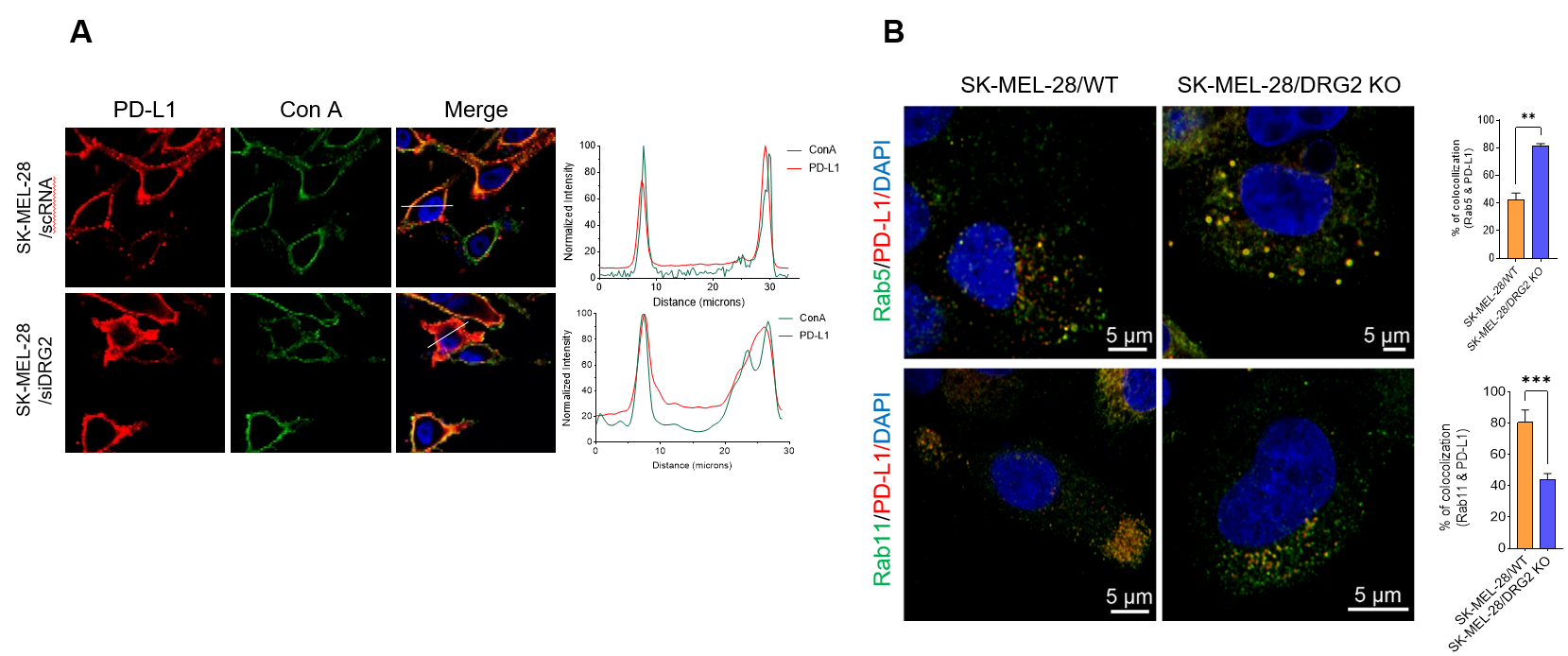


**Supplementary Fig. S5 DRG2-depleted human melanoma cells show defects in recycling of endosomal PD-L1 and accumulation of PD-L1 at Rab5-endosomes**. **Related to Fig. 4.** **A** DRG2 deficiency decreases surface membrane PD-L1 in SK-MEL-28 human melanoma cells. IFN-γ-treated SK-MEL-28/scRNA and SK-MEL-28/siDRG2 cells were stained with ConA. After fixation they were incubated with anti-PD-L1 antibody. Line graphs represent linear pixel values across cells. **B** DRG2 deficiency decreases the PD-L1 level in Rab11-endosomes but increases the PD-L1 level in Rab5-endosomes. Representative confocal images of SK-MEL-28/WT and SK-MEL-28/DRG2 KO cells incubated with anti-DRG2, anti-PD-L1, anti-Rab5, and anti-Rab11 at 30 min after IFN-γ treatment. Blue, DAPI staining. Graph represents Pearson’s *R*(*r*) between PD-L1 and Rab5 or Rab11. Values are mean ± SD from two experiments, with 20 different cells per group per experiment. ***p* < 0.01; ****p* < 0.001.


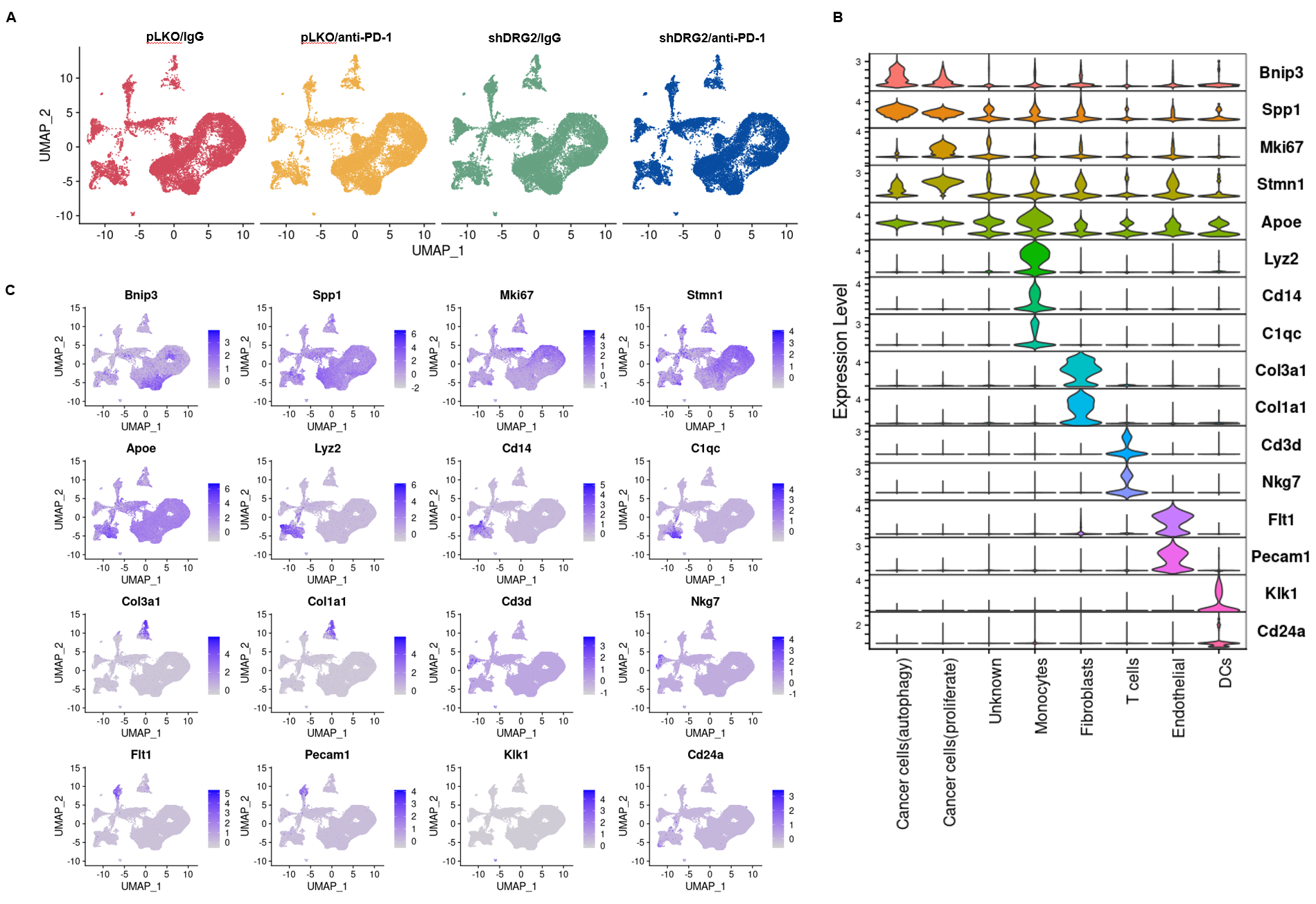


**Supplementary Fig. S6 Cell types detected in melanoma tumors based on single-cell RNA-Seq profiling of 62,956 cells**. **Related to Fig. 6.** **A** UMAP of cells from B16F10/pLKO or B16F10/shDRG2 tumors treated with negative control (IgG) or anti-PD-1. **B** Violin plots showing the log10 expression of marker genes for each cell cluster. **C** UMAP of 62,956 cells showing the expression of marker genes for each cell cluster.


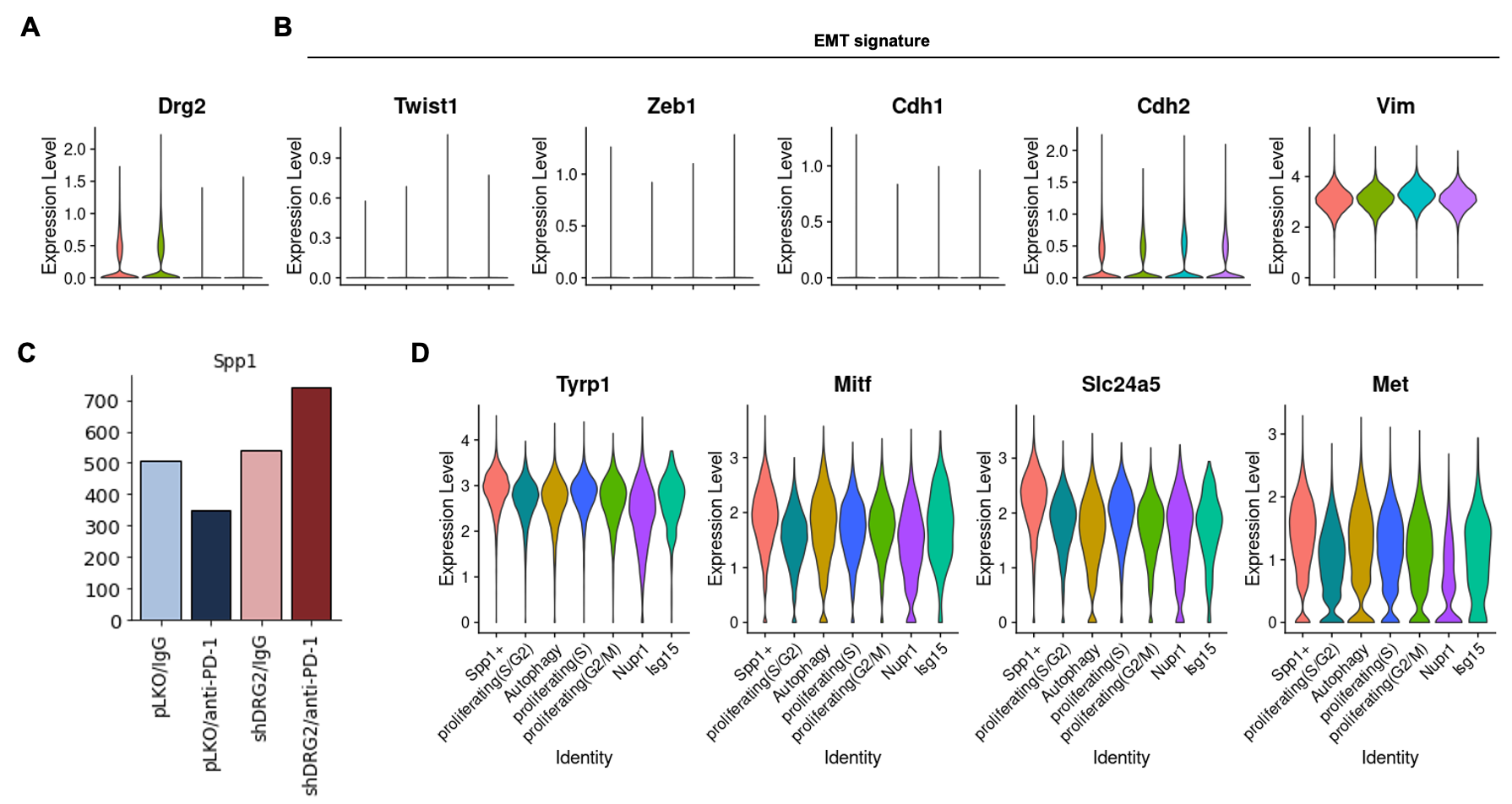


**Supplementary Fig. S7 Expression of EMT-related genes in cancer cells and differentially expressed genes for “Spp1+” subcluster associated with melanocyte differentiation. Related to Fig. 6. A** Violin plots showing the expression of (**A**) DRG2 and **B** EMT-related genes in cancer cells within B16F10/pLKO or B16F10/shDRG2 tumors treated with IgG or anti-PD-1. **C** Normalized count of Spp1 for each sample. **D** Violin plots showing the log2 expression of the 4 genes highly expressed in Spp1+ cancer cell cluster and associated with melanocyte differentiation (*Tyrp1*, *Mitf*, *Slc24a5*, and *Met*).


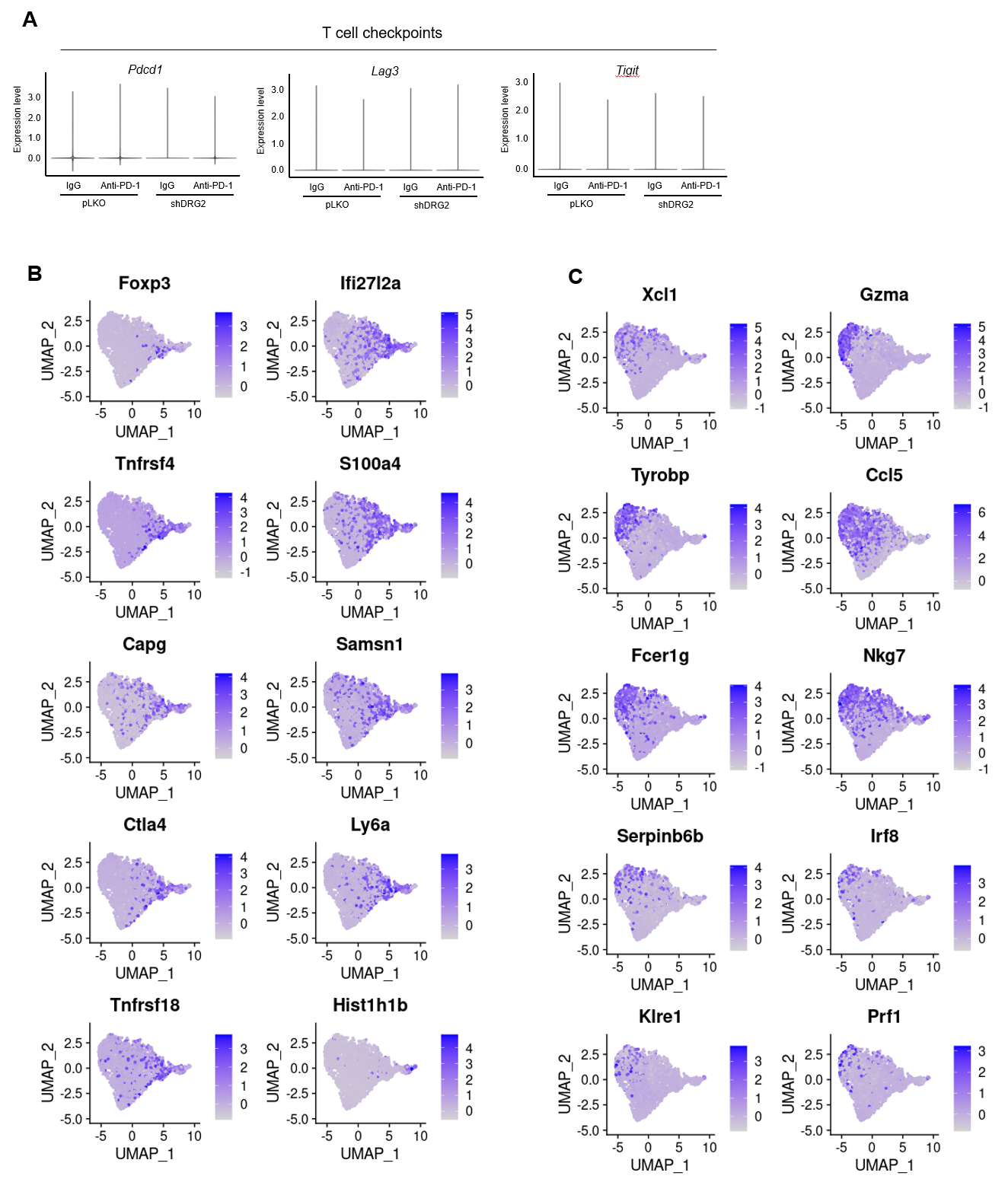


**Supplementary Fig. S8 Expression of immune checkpoint genes in T cells and the top-10 most differentially expressed genes for T cell subcluster 2 and 3 across T cell clusters. Related to Fig. 7. A** Violin plots showing the expression of immune checkpoint genes, including *Pdcd1*, *Lag3*, and *Tigit* in T cells cancer cells within B16F10/pLKO or B16F10/shDRG2 tumors treated with negative control (IgG) or anti-PD-1. **B,C** UMAP of 2,341 T cells showing the expression of the top-10 most differentially expressed genes for (**B**) T cell subcluster 2 and (**C**) T cell subcluster 3 across the T cell cluster.
