# Supplementary material for: DRG2 is required for surface localization of PD-L1 and the efficacy of anti-PD-1 therapy
Source: Cell Death Discov. 2024 May 27;10:260. doi: 10.1038/s41420-024-02027-x (PMC11130180; doi:10.1038/s41420-024-02027-x)
Supplement: Supplementary file 5 — Table S4 [file 41420_2024_2027_MOESM5_ESM.docx]

Table S4. PCR primers used in this study

| Primers |  | Sequences(5'-3') |
| --- | --- | --- |
| Human β-actin | F | CACCATTGGCAATGAGCGGTTC |
|  | R | AGGTCTTTGCGGATGTCCACGT |
| Human DRG2 | F | ACAGACGCCATCATTCTCCG |
|  | R | CACACCAGGGCGTACTTGAA |
| Human IRF1 | F | AAGCATGGCTGGGACATCAA |
|  | R | TGCTTTGTATCGGCCTGTGT |
| Human PD-L1 | F | TTTGCTGAACGCCCCATACA |
|  | R | TCCAGATGACTTCGGCCTTG |
| Mouse β-actin | F | CATTGCTGACAGGATGCAGAAGG |
|  | R | TGCTGGAAGGTGGACAGTGAGG |
| Mouse CD70 | F | CCGCACACAGCTGAGTTACA |
|  | R | CTCTGGTCCGTGTGTGAAGG |
| Mouse CD80 | F | TCAATACGACTCGCAACCACA |
|  | R | GAGGGTCTTCTGGGGGTTTT |
| Mouse CD86 | F | AAAGAGGAGCAAGCAGACGC |
|  | R | CATGGTGCATCTGGGGTCCAT |
| Mouse DRG2 | F | TGGAACCATCCAAATCCGCC |
|  | R | CAAGAAGGTGGACTTACCCACA |
| Mouse Gal9 | F | GTGCAGTTCTCTCAGCCAGT |
|  | R | GTGGGCAGGACGAAAGTTCT |
| Mouse IRF1 | F | GAAAGTCCAAGTCCAGCCGA |
|  | R | GCTGTGGTCATCAGGTAGGG |
| Mouse OX40L | F | AGAAGACGCTAAGGCTGGTG |
|  | R | GCCGGAGAGGAAGAGAGTTG |
| Mouse PD-L1 | F | TGCTGCATAATCAGCTACGG |
|  | R | TCCACGGAAATTCTCTGGTT |
| Mouse PD-L2 | F | TTATTCACCGTGACAGCCCC |
|  | R | AGTGCATTCTCTGCGGTCAA |
| Mouse 4-1BBL | F | ACCTGGGTACCCGAGAGAAT |
|  | R | GTAGCTTGGCGAACACAGGA |
