# Supplementary material for: DRG2 is required for surface localization of PD-L1 and the efficacy of anti-PD-1 therapy
Source: Cell Death Discov. 2024 May 27;10:260. doi: 10.1038/s41420-024-02027-x (PMC11130180; doi:10.1038/s41420-024-02027-x)
Supplement: Supplementary file 6 — Original data files [file 41420_2024_2027_MOESM6_ESM.pptx]

## Slide 1
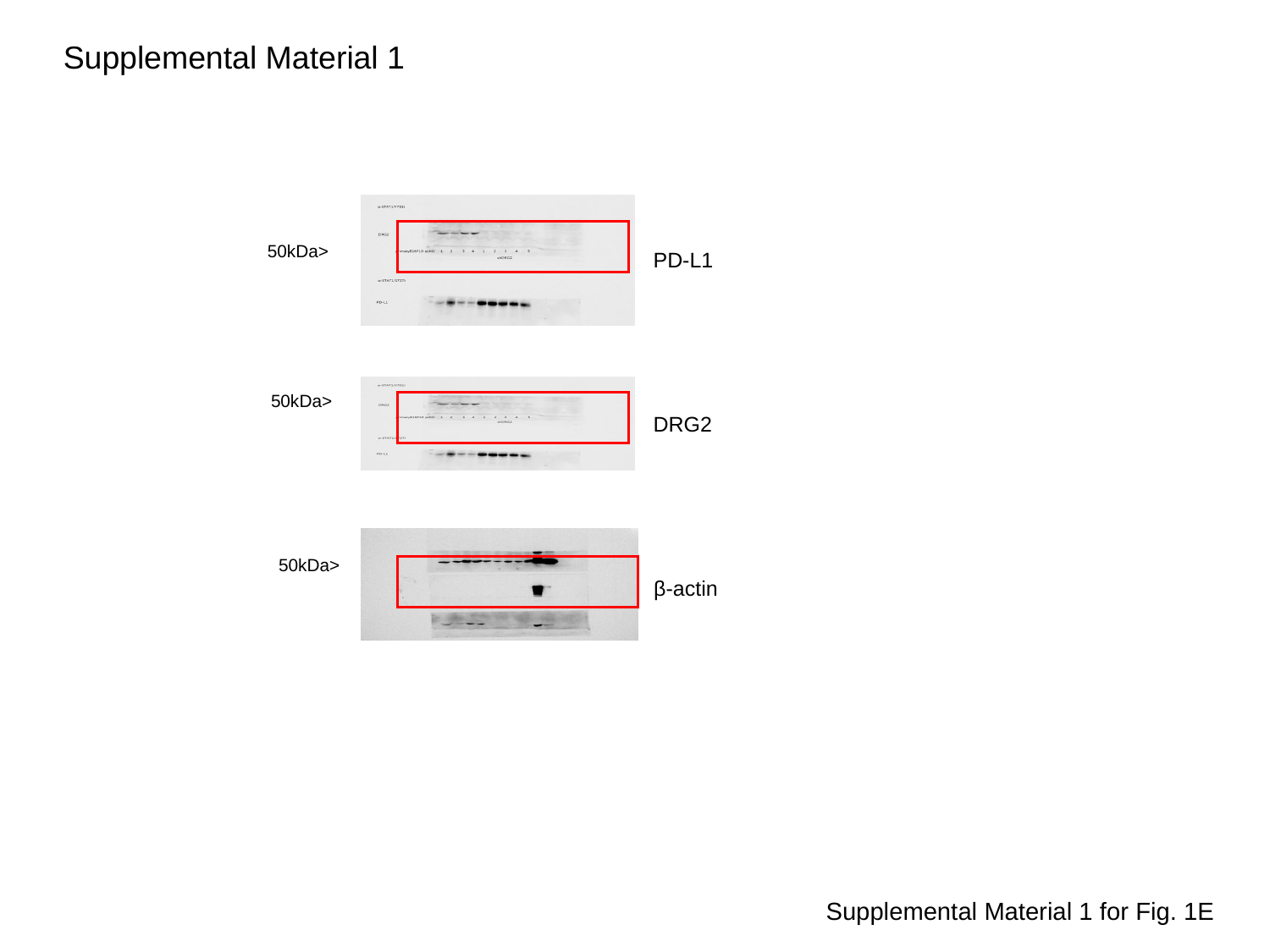

Supplemental Material 1
50kDa>
PD-L1
50kDa>
DRG2
50kDa>
β-actin
Supplemental Material 1 for Fig. 1E

## Slide 2
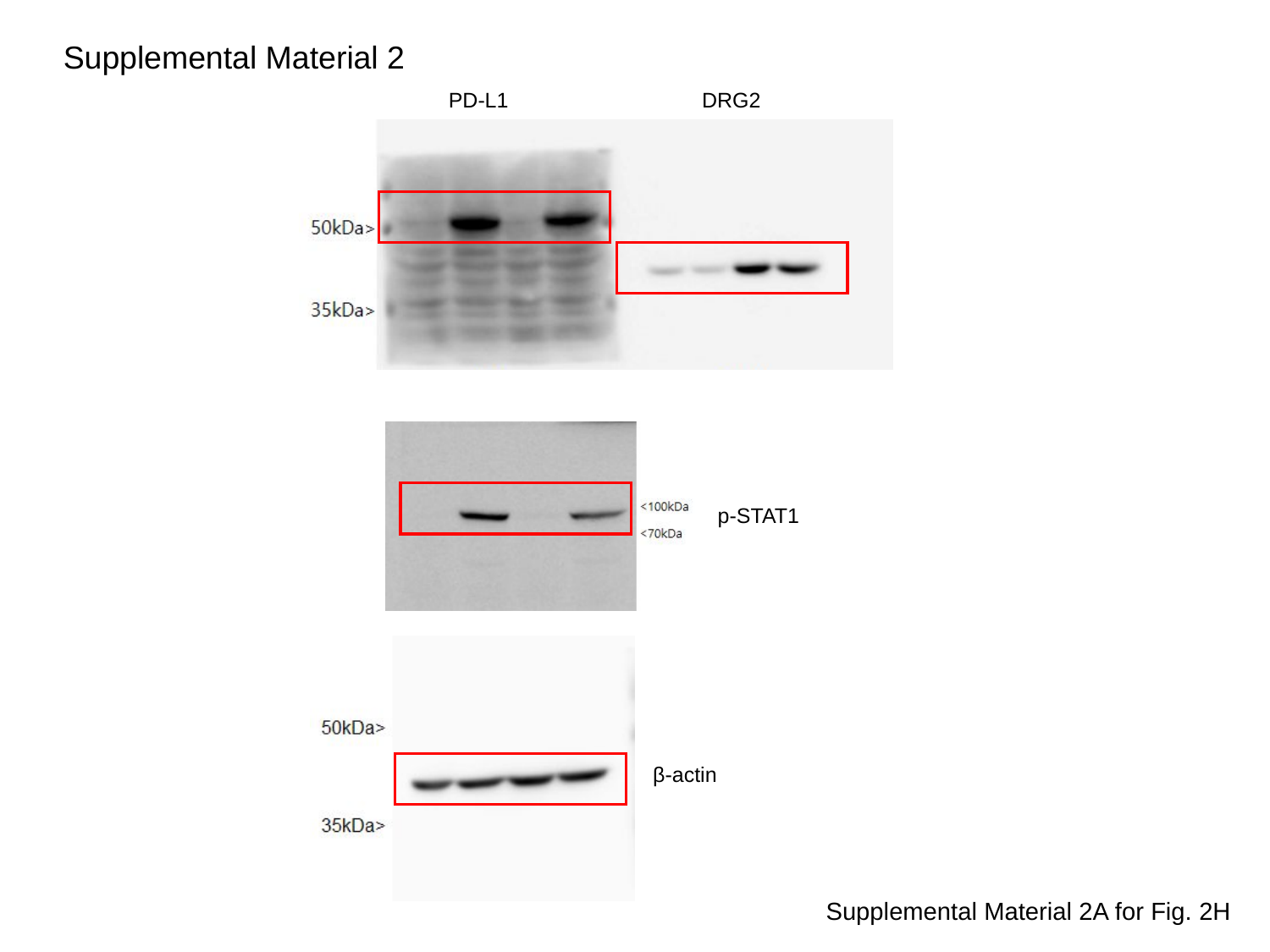

Supplemental Material 2
PD-L1
DRG2
p-STAT1
β-actin
Supplemental Material 2A for Fig. 2H

## Slide 3
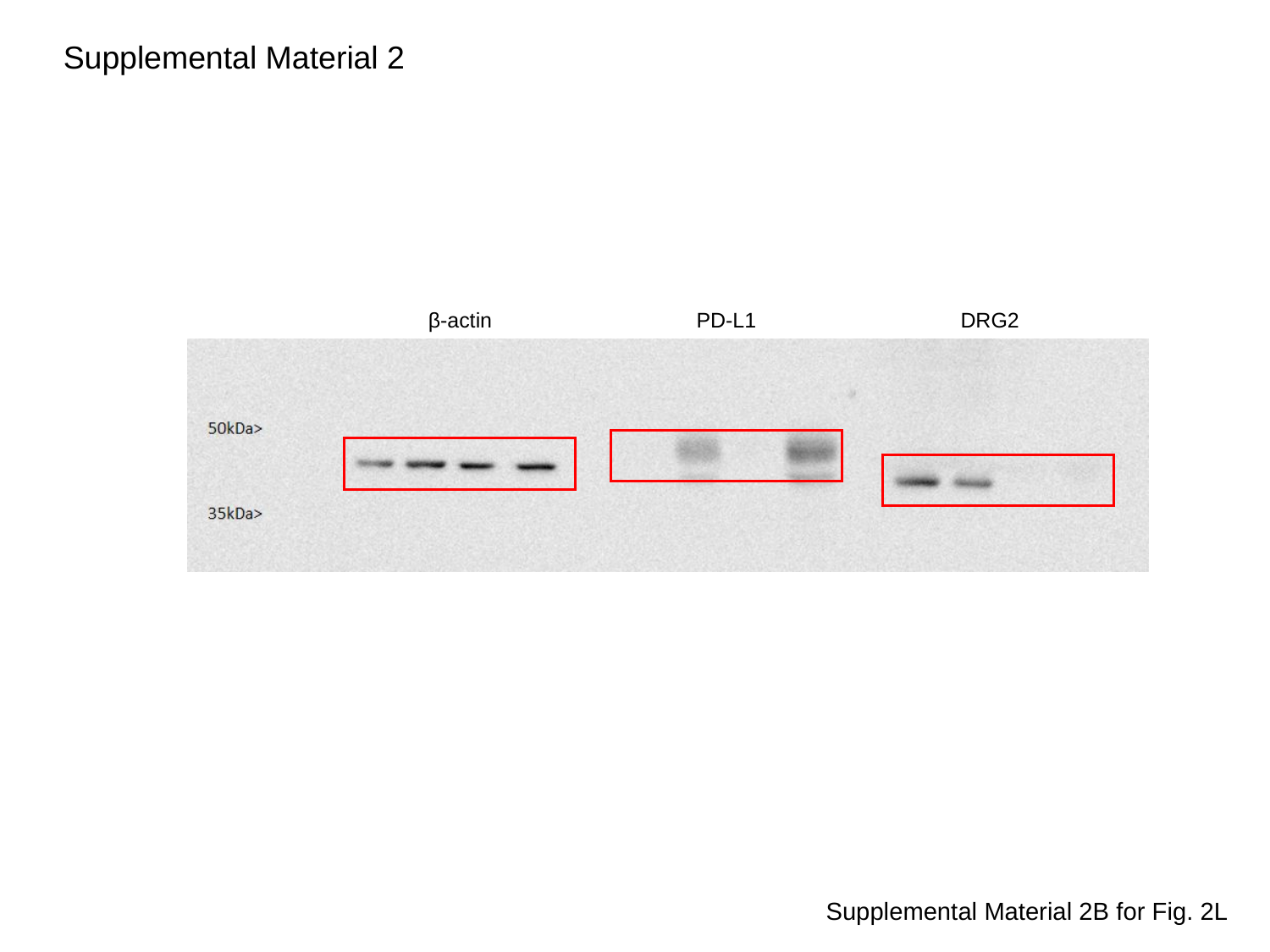

Supplemental Material 2
PD-L1
DRG2
β-actin
Supplemental Material 2B for Fig. 2L

## Slide 4
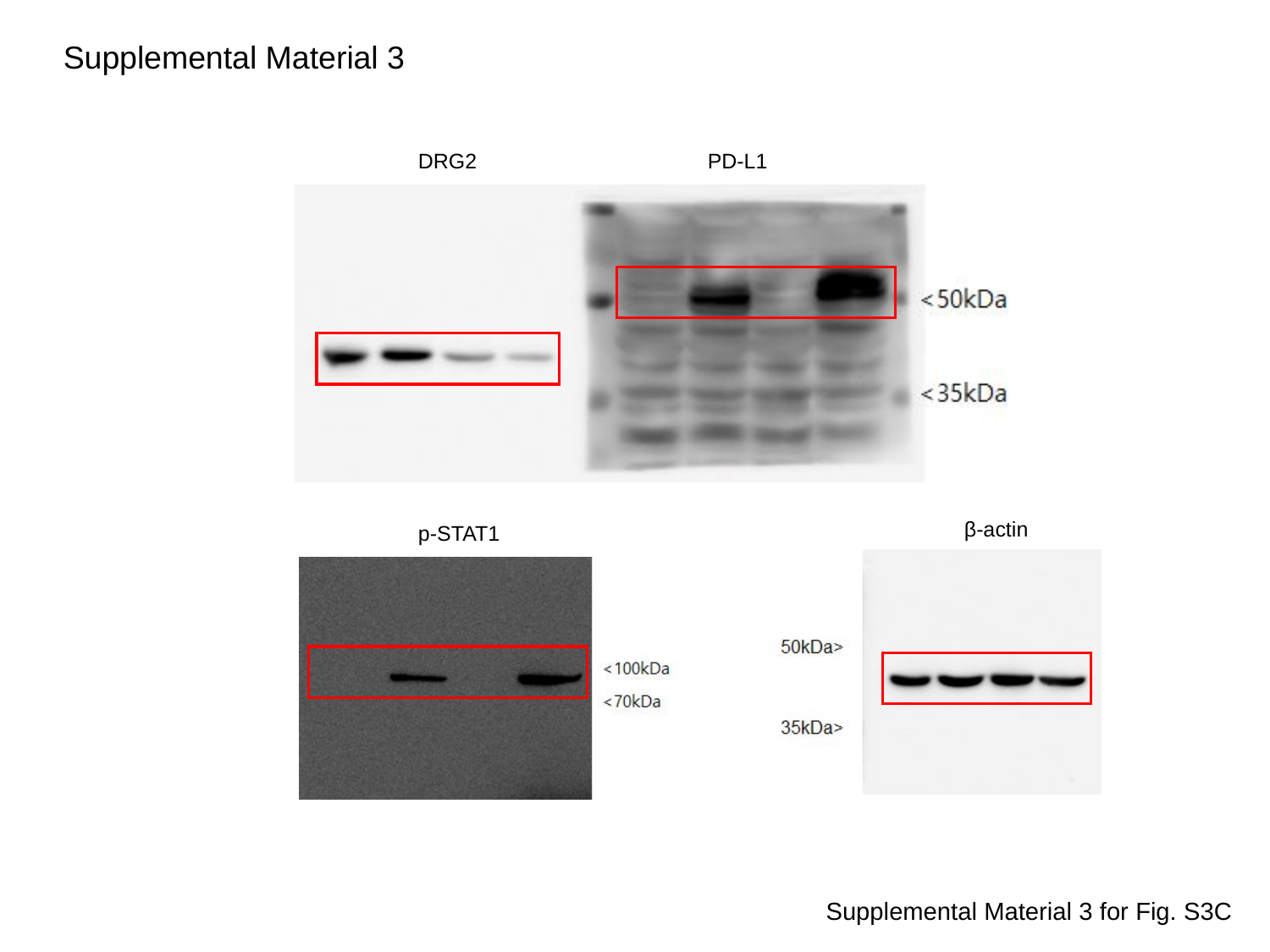

Supplemental Material 3
DRG2
PD-L1
β-actin
p-STAT1
Supplemental Material 3 for Fig. S3C

## Slide 5
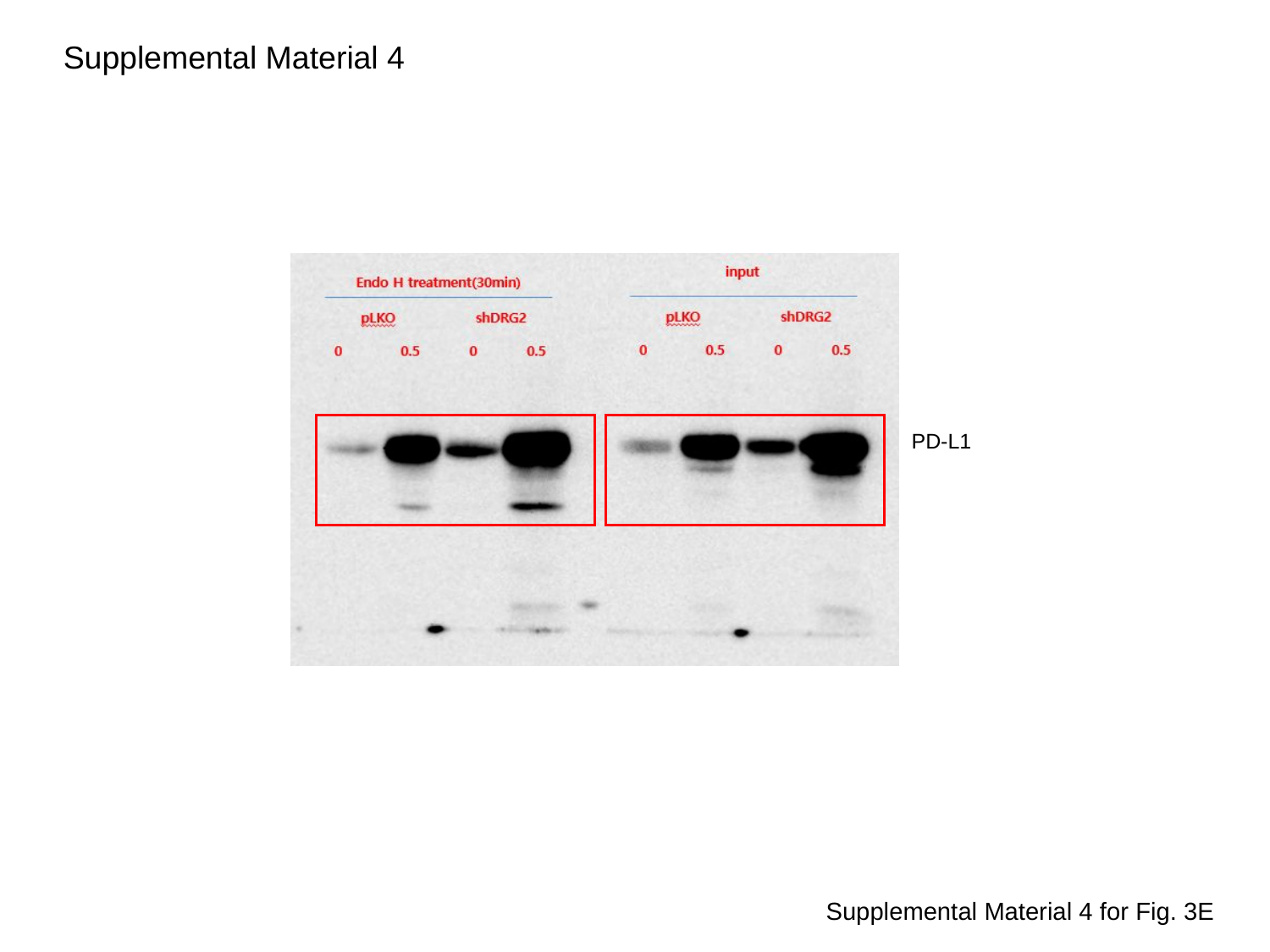

Supplemental Material 4
PD-L1
Supplemental Material 4 for Fig. 3E
